# DUSP1 and SOX2 expression determine squamous cell carcinoma of the salivary gland progression

**DOI:** 10.1038/s41598-024-65945-x

**Published:** 2024-07-01

**Authors:** Lucía Acero-Riaguas, Ana Belén Griso-Acevedo, Alejandro SanLorenzo-Vaquero, Blanca Ibáñez-Herrera, Sara María Fernandez-Diaz, Marta Mascaraque, Rocío Sánchez-Siles, Iván López-García, Carlos Benítez-Buelga, Elena Ruiz Bravo-Burguillos, Beatriz Castelo, José Luis Cebrián-Carretero, Rosario Perona, Leandro Sastre, Ana Sastre-Perona

**Affiliations:** 1 Laboratory of Translational Research in Maxillofacial Surgery and Head and Neck Cancer, IdiPAZ, 28046 Madrid, Spain; 2 Medical Oncology Department, University Hospital La Paz, 28046 Madrid, Spain; 3 Oral and Maxillofacial Surgery Department, University Hospital La Paz, 28046 Madrid, Spain; 4 Instituto de Investigaciones Biomédicas CSIC/UAM and CIBER de Enfermedades Raras (CIBERER), 28029 Madrid, Spain; 5 Instituto de Salud Carlos III and CIBER de Enfermedades Raras (CIBERER), 28029 Madrid, Spain

**Keywords:** Head and neck cancer, Cell growth

## Abstract

Salivary gland squamous cell carcinomas (SG-SCCs) constitute a rare type of head and neck cancer which is linked to poor prognosis. Due to their low frequency, the molecular mechanisms responsible for their aggressiveness are poorly understood. In this work we studied the role of the phosphatase DUSP1, a negative regulator of MAPK activity, in controlling SG-SCC progression. We generated *DUSP1* KO clones in A253 human cells. These clones showed a reduced ability to grow in 2D, self-renew in ECM matrices and to form tumors in immunodeficient mice. This was caused by an overactivation of the stress and apoptosis kinase JNK1/2 in *DUSP1*^−/+^ clones. Interestingly, RNAseq analysis revealed that the expression of SOX2, a well-known self-renewal gene was decreased at the mRNA and protein levels in *DUSP1*^−/+^ cells. Unexpectedly, CRISPR-KO of *SOX2* did not recapitulate *DUSP1*^−/+^ phenotype, and *SOX2*-null cells had an enhanced ability to self-renew and to form tumors in mice. Gene expression analysis demonstrated that *SOX2*-null cells have a decreased squamous differentiation profile -losing TP63 expression- and an increased migratory phenotype, with an enhanced epithelial to mesenchymal transition signature. In summary, our data indicates that DUSP1 and SOX2 have opposite functions in SG-SCC, being DUSP1 necessary for tumor growth and SOX2 dispensable showing a tumor suppressor function. Our data suggest that the combined expression of SOX2 and DUSP1 could be a useful biomarker to predict progression in patients with SG-SCCs.

## Introduction

Salivary gland malignancies are rare types of cancers that represent 5.7% of all head and neck cancers^1^. These types of cancers develop from the three types of salivary glands: parotid (59–81% of cases), submandibular (6–21%) and minor (7–22%) salivary glands^2,3^. When transformation of the tissue occurs, it can give rise to up to 20 histologically distinct cancer subtypes recognized by the World Health Organization (WHO)^4^. The high histological heterogeneity of these cancers can be explained by the composition of the salivary glands, which contain several cell types, including myoepithelial, acinar, ductal, basal, and epidermoid cells^5^. Although each cancer subtype is thought to arise from a specific cell type, tumors are frequently composed by a combination of lineages. However, it is still unclear which is the contribution of each cell type to tumor progression and aggressiveness. The low frequency of salivary gland cancers combined with their high diversity, has difficulted their characterization at a molecular level. As a result, there are no proper diagnostic tools that can effectively predict treatment options and patient progression.

A particularly relevant type of salivary gland cancer is the squamous cell carcinoma subtype (SG-SCC)^6,7^. SG-SCCs represent 0.3–6.9% of all salivary gland neoplasms. The origin of this cancer is controversial, since it can manifest as a metastasis from a cutaneous SCC primary tumor from the head and neck region, but in the cases where there is no record of a primary tumor, it is thought to originate de novo from the salivary glands^6^. Regardless of the origin, the presence of SG-SCC is linked to poor outcome^8^. Therefore, understanding the molecular drivers behind these cancers can be useful to develop new therapeutic interventions.

In mice, SG-SCCs can originate from K14-positive basal cells^9,10^, through the constitutive activation of the Wnt pathway and the inhibition of BMP signaling. This mouse model recapitulated the histology of human cancers and allowed the description of key cancer cell populations and their transcriptional programs. Using pseudotime analysis of scRNAseq data, Praktiknjo et al. calculated the trajectory of cancer cells, predicting a transition from normal basal cells to basal cancer cells, which can further derive in cells with cancer stem cell (CSC) properties that will undergo differentiation into a luminal cell subtype, more represented in advanced tumors. K14-positive basal cells express epithelial programs driven by *Trp63*^5^, which has been shown to be critical to sustain salivary gland homeostasis. Due to the epithelial nature of these cells, we wondered how known drivers of epithelial carcinogenesis could affect the evolution of SG-SCCs.

In this work we aimed to explore how the imbalance of MAPK activity can affect SG-SCC progression. MAPK signaling pathways are well known to regulate SCC progression. Particularly the implication of the HRAS-ERK pathway is very well understood in cutaneous SCC, where HRAS oncogene is frequently mutated and used as a tumor driver in mouse models^11^. On the other hand, JNK1 kinase has been shown to promote apoptosis in cutaneous SCC, while JNK2 promotes carcinogenesis^12^. But to our knowledge, there is no literature on the function of the different MAPKs pathways in the specific context of SG-SCC.

To start to study their function, we were particularly interested in the role of DUSP1, a phosphatase that controls the activity of three MAPK members ERK1/2, JNK1/2 and p38^13^. This phosphatase has a context-dependent role in cancer depending on the main target, having a tumor suppressor role when it preferentially inhibits the pro-mitotic kinase ERK, or an oncogenic role when it acts inhibiting the stress and pro-apoptotic kinases p38 and JNK. We used CRISPR/Cas9 to delete DUSP1 in the A253 cell line and demonstrated a preferential oncogenic role of DUSP1 by inhibiting JNK activity. As a result, we found that *DUSP1*^−/+^ cells lose stemness properties and are not able to form tumors. We also identified that the expression of SOX2, a key stemness transcription factor^14^, was decreased in *DUSP1*^*-/*+^ cells. To probe if SOX2 downregulation was responsible for the *DUSP1*^*-/*+^ phenotype, we applied CRISPR to mutate *SOX2*. Surprisingly, we uncovered that rather than impairing self-renewal, SOX2 loss accelerated stemness and tumoral growth. We went on to demonstrate that loss of SOX2 promotes a switch from epithelial to mesenchymal phenotype, with an increase in luminal phenotype. In summary, we demonstrated the opposite role of DUSP1 and SOX2 in driving SG-SCC growth, and we propose the use of the combined expression of SOX2 and DUSP1 as a predictor of SG-SCC prognosis.

## Results

### DUSP1 controls cell growth and self-renewal in SG-SCCs

**Figure 1:**
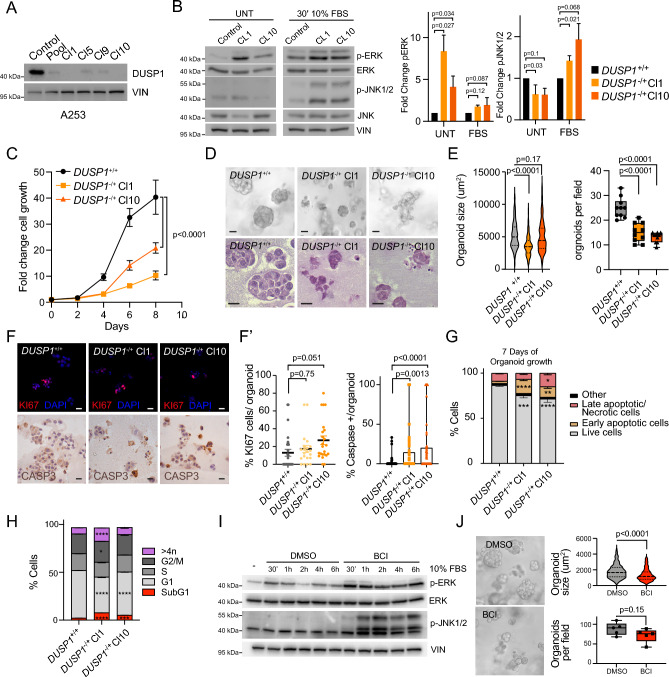
DUSP1 loss decreases cell growth and self-renewal in SG-SCC. (**A**) Western blot illustrating DUSP1 expression in A253 control cells and *DUSP1*^−/+^ pool of cells and clones (Cl1, 5, 9 and 10). (**B**) Left, Western blot illustrating ERK and JNK phosphorylation in A253 control and DUSP1 KO clones in basal medium (0.5% FBS) or stimulated 30 min with 10% FBS. Right, Bar graphs showing pERK/ERK and pJNK/JNK ratio quantifications (n = 3, t-test). (**C**) Graph illustrating the fold change growth of control (*DUSP1*^+/+^) and DUSP-1KO clones (n = 3, Two-way ANOVA). (**D**) Brightfield (upper panel) or H&E stains micrographs of organoid sections (lower panel) illustrating the size and shape of *DUSP1*^+/+^ and *DUSP1*^−/+^ clones after 7 days growing in BME. (**E**) Violine plot illustrating organoid size (Left) and boxplot illustrating organoids/field in *DUSP1*^+/+^ and *DUSP1*^−/+^ clones (One-way ANOVA). (**F**) Representative micrographs of organoid sections stained with KI67 (red, upper panel) or Casp3 (lower panel) of *DUSP1*^+/+^ and *DUSP1*^−/+^ clones. (**F’**) Scatter plot and box plot illustrating the percentage of KI67 or Caspase 3 positive cells per organoid (one-way ANOVA). (**G**) Graph illustrating the percentage of apoptotic of live cells in organoids (*p = 0.05–0.01; **p < 0.01; ***p < 0.001 and ****p < 0.0001; n = 3, two-way ANOVA). (**H**) Graph illustrating the percentage of cell through the cell cycle (*p = 0.05–0.01; **p < 0.01; ***p < 0.001 and ****p < 0.0001; n = 3, two-way ANOVA). (**I**) Representative western blot illustrating the activation of pERK and pJNK in response to BCI (5 uM) over time. (**J**) Left, Representative brightfield micrographs illustrating A253 organoids treated with DMSO or BCI (5 uM) for 7 days. Right, Violine plot illustrating organoid size (upper) and boxplot illustrating organoids/field (lower) in DMSO of BCI treat A253 cells (n = 3, *t*-test). Scale bars, 50 µm.

To investigate the potential role of DUSP1 in the context of salivary gland squamous cell carcinomas (SG-SCCs), we applied the CRISPR/Cas9 system to delete exon 2 of *DUSP1* in A253 human cells, derived from a primary SG-SCC. We were only able to obtain heterozygous deletions of DUSP1 (Figure Supp. 1A), but we obtained two clones (Cl1 and Cl10) that showed no expression of DUSP1 at the protein level in comparison to control cells, and therefore selected them to continue our studies (Fig. 1A, Figure Supp 1B). Analysis of the MAPK pathways regulated by DUSP1 showed that ERK was more activated in *DUSP1*^−/+^ clones in basal conditions, while both JNK1/2 and ERK were more activated in *DUSP1*^−/+^ clones in response to serum (Fig. 1B). This would suggest that DUSP1 inhibition could trigger both proliferative and stress MAPKs. To distinguish the prevalent effect, we measured the consequence of *DUSP1* deletion in cell growth, identifying that *DUSP1*^-/+^ clones have decreased growth capacity (Fig. 1C). Next, we analyzed the self-renewal capacity of *DUSP1*^−/+^ clones by performing organoid formation assays in ECM matrix (Fig. 1D). After 7 days, we detected a significantly smaller organoid size in Cl1 and a decreasing tendency in Cl10, with a significant reduction on organoid number in both clones, suggesting that *DUSP1*^*-/*+^ clones have a decreased self-renewal capacity (Fig. 1E). Unexpectedly, we detected similar or even higher KI67-proliferative cells or pH3-positive mitotic cells (Fig. 1F, F’ upper panels, Figure Supp 1C) in organoids derived from both clones in comparison to control cells. To determine if the decreased size of *DUSP1*^*-/*+^ cells could be due to apoptosis, we stained the organoids with an active-Caspase-3 antibody detecting an increased number of single cells expressing Caspase-3 on *DUSP1*^*-/*+^ organoids (Fig. 1 F, F’ lower panels). This result was corroborated performing AnnexinV/7ADD staining and flow cytometry analysis on organoids, which indicated that *DUSP1*^−/+^ organoids have higher percentage of early and late apoptotic cells, and therefore, lower number of live cells (Fig. 1G). In agreement with this data, we also detected lower G1 and higher subG1 damage cells in *DUSP1*^−/+^ organoids by doing cell cycle assays (Fig. 1H). This data suggests that *DUSP1*^−/+^ organoids might not form because the cells could not survive in 3D conditions and die from apoptosis.

To reproduce the effect of DUSP1 loss, we treated wild-type cells with BCI, a small molecule that inhibits DUSP1 activity^15^. By western blot we could demonstrate that BCI effectively induced both ERK and JNK1/2 activation (Fig. 1I). We added BCI to A253 organoids since day 1 of the organoid culture and measured the effects on day 7, detecting a significant decrease in organoid size and a tendency to a decreased organoid number (Fig. 1J). This data indicated that chemical inhibition of DUSP1 recapitulates the effects of *DUSP1* genetic ablation. Overall, this result suggests that DUSP1 loss leads to an increase in apoptosis in SG-SCCs.

### DUSP1 deletion impairs tumor growth in SG-SCC

**Figure 2:**
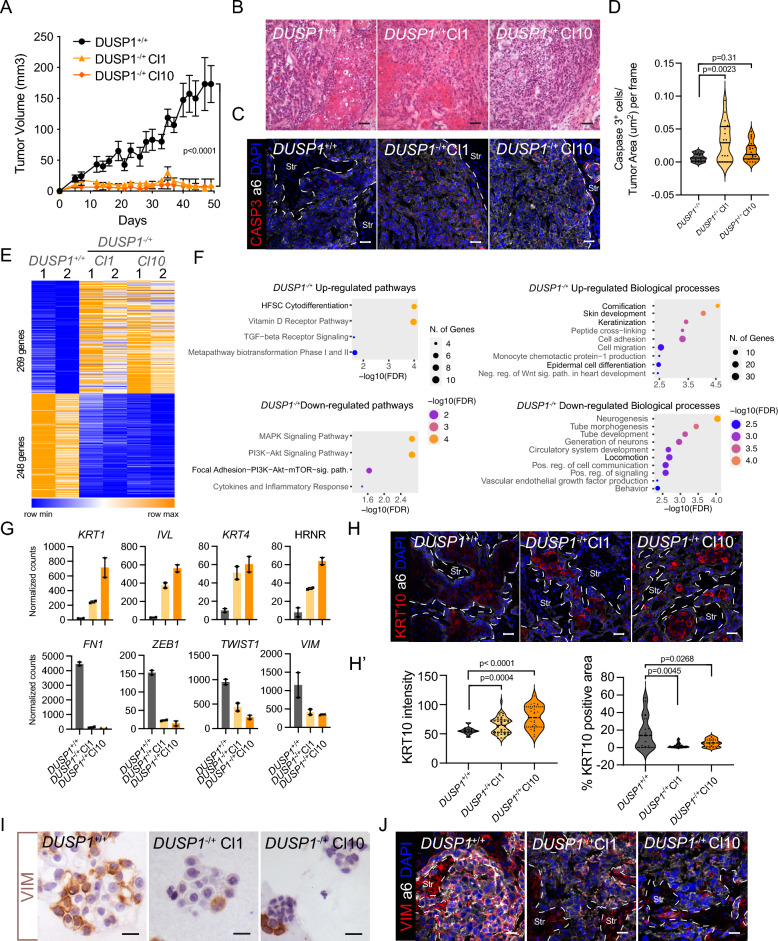
DUSP1 loss impairs tumor growth and triggers squamous differentiation in SG-SCCs. (**A**) Tumor growth rates after intradermal injection of 100,000 control (black) and *DUSP1*^−/+^ (orange) human A253 cells (mean ± SEM, n = 6 transplants, two-way ANOVA). (**B**) H&E stains of sections from control of *DUSP1*^−/+^ tumors. (**C**) Confocal micrographs illustrating Caspase 3 positivity (red) within a6 integrin positive (white) tumor cells. (**D**) Violine plot illustrating the number of Caspase 3 positive cells per tumor area in *DUSP1*^+/+^ and *DUSP1*^−/+^ tumors (n = 3, One-way ANOVA). (**E**) Heatmap illustration the DEG between *DUSP1*^+/+^ and *DUSP1*^−/+^ cells. (**F**) Graph showing the pathways (left) or gene ontology biological processes (right) enriched or depleted in *DUSP1*^−/+^ cells. In bold are highlighted the most relevant findings. (**G**) Bar graphs showing the increased (upper) expression (Normalized counts) of squamous genes and decreased expression (lower) of mesenchymal genes in *DUSP1*^−/+^ cells. (**H**) Confocal micrographs illustrating KRT10 positivity (red) within a6 integrin positive (white) tumor cells. (**H’**) Violin plots quantifying the intensity (right panel) or area (right panel) of KRT10 on *DUSP1*^+/+^ and *DUSP1*^−/+^ tumors (One-way ANOVA). (**I**) Brightfield (left) and (**J**) confocal (right) micrographs of Vimentin (brown or red) staining on organoid or tumor sections respectively from *DUSP1*^+/+^ and *DUSP1*^−/+^ cells. a6 integrin staining (white) demarcates the boundary between tumor epithelial cells and tumor stroma (Str). Scale bars, H = 50 µm.

To test the function of DUSP1 in tumor growth, we transplanted *DUSP1* control and KO clones intradermally in immunodeficient mice (nu/nu). Although all the tumors grew initially, *DUSP1*^*-/*+^ tumors remained small and did not evolve over time in comparison to control tumors (Fig. 2A). H&E staining showed that *DUSP1*^−/+^ tumors presented as disorganized with very few squamous structures in comparison with control tumors (Fig. 2B). *DUSP1*^−/+^ tumors showed an increased number of Caspase 3-positive cells (Fig. 2C, D), but surprisingly, *DUSP1*^−/+^ tumors still showed no difference in the expression of proliferative markers KI67 and pH3 (Figure Supp 2A, B). To better understand this phenotype, we subjected cells from control and *DUSP1*^−/+^ clones to RNAseq. We performed differential gene expression (DGE) analysis comparing control and *DUSP1*^−/+^ clones and chose only the genes that concordantly changed in both 1 and 10 *DUSP1*^*-/*+^clones (Figure Supp 2C, Table Supp. 1). This analysis revealed 269 genes upregulated and 248 genes downregulated in both *DUSP1*^−/+^ clones (Fig. 2E). Increased genes represented mainly processes related to keratinization (Cornification, epidermal cell differentiation) with genes such as *FGL*, *IVL*, *HRNR*, *KRT1* or *KRT4* (Fig. 2F, G) being increased in the absence of *DUSP1*. On the other hand, downregulated genes included pathways related with cell migration such as focal-adhesion kinase and PI3K-AKT, and biological processes such as locomotion (Fig. 2F). Genes of these pathways included well-known regulators of epithelial to mesenchymal transition (EMT) such as *ZEB1* and *TWIST1* and their regulated genes *FN1* and *VIM* (Fig. 2G). We validated the increased expression of keratinization related genes by qPCR, demonstrating an increased expression of *KRT1*, *IVL*, *FLG* or *KRT4* (Figure Supp 2D). Additionally, we measured KRT10 protein levels detecting higher expression of KRT10 in *DUSP1*^-/+^ tumors in comparison to the control (Fig. 2H). Interestingly, the areas occupied by KRT10 positive cells were smaller in *DUSP1*^−/+^ cells, confirming our original observation of decreased keratin pearls. This may suggest that in the absence of DUSP1, cells differentiate expressing higher levels of KRT, but fail to expand and form keratin pearls.

Vimentin (VIM) staining in organoid sections, as well as in tumor sections demonstrated a marked reduction in vimentin expression, specifically in cancer cells (Fig. 2I, J) from both *DUSP1*^−/+^ clones, validating the previous results. Altogether, these data show that *DUSP1* deficiency promotes a more differentiated, squamous phenotype.

### SOX2 does not mediate DUSP1 phenotype

**Figure 3:**
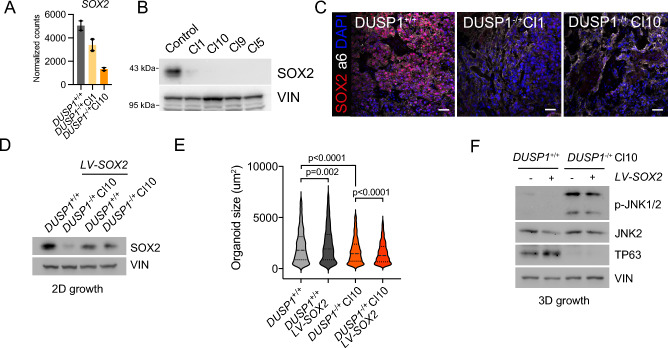
SOX2 expression is lost in DUSP1^−/+^ but its over-expression cannot rescue DUSP1^−/+^ phenotype. (**A**) Gene expression of SOX2 in *DUSP1*^+/+^ and *DUSP1*^−/+^ cells. (**B**) Western blot showing SOX2 protein expression in *DUSP1*^+/+^ and *DUSP1*^−/+^ cells. (**C**) Confocal micrographs showing SOX2 expression (red) within a6-integrin (white) positive cancer cells. (**D**) Western blot illustrating the over expression of SOX2. (**E**) Violine plot illustrating organoid size in *DUSP1*^+/+^ and *DUSP1*^−/+^ clone with or without SOX2 OE (One-way ANOVA). (**F**) Western blot from *DUSP1*^+/+^ and *DUSP1*^−/+^ organoids with or without SOX2 OE illustrating JNK activity. Scale bars, 100 µm.

Since *DUSP1* KO clones had reduced self-renewal capacity in vitro and in vivo, we looked for known self-renewal transcription factors downregulated in both *DUSP1*^*-/*+^ clones, identifying SOX2 (Fig. 3A, Figure Supp 2D). We validated that SOX2 protein was downregulated to almost undetectable protein levels in all *DUSP1*^−/+^ clones that we generated (Fig. 3B), showing that this effect was independent of the clone. This was confirmed using BCI treatment, which also decreased SOX2 protein levels (Figure Supp. 2E). Finally, we observed a strong decrease in SOX2 protein level in *DUSP1*^−/+^ tumors in comparison with the control (Fig. 3C). These data suggest that *DUSP1*^−/+^ tumor cells are more differentiated, and this could be due to a loss of the self-renewal transcription factor (TF) SOX2 as it was described in the cutaneous SCC (cSCC) context^14^.

To distinguish if the decreased SOX2 expression could be driving *DUSP1*^−/+^ phenotype, we rescued SOX2 expression on *DUSP1*^−/+^ Cl10 using a lentiviral construct. After we confirmed the correct increase in SOX2 protein on *DUSP1*^−/+^ Cl10 (Fig. 3D), we performed organoid cultures and measured their growth at the endpoint of the experiment. While control organoids grew significantly bigger with SOX2 OE, *DUSP1*^−/+^ Cl10 organoids grew even smaller upon SOX2 OE (Fig. 3E). Western blot analysis of organoid protein lysate demonstrated that *DUSP1*^−/+^ Cl10 organoids express higher levels of pJNK than controls, and this could not be rescued by SOX2 OE (Fig. 3F). We concluded that increased JNK activation rather than SOX2 loss of expression was responsible of the cell death induced after DUSP1 loss.

### SOX2 silencing enhances SG-SCC cell proliferation and tumor growth

**Figure 4:**
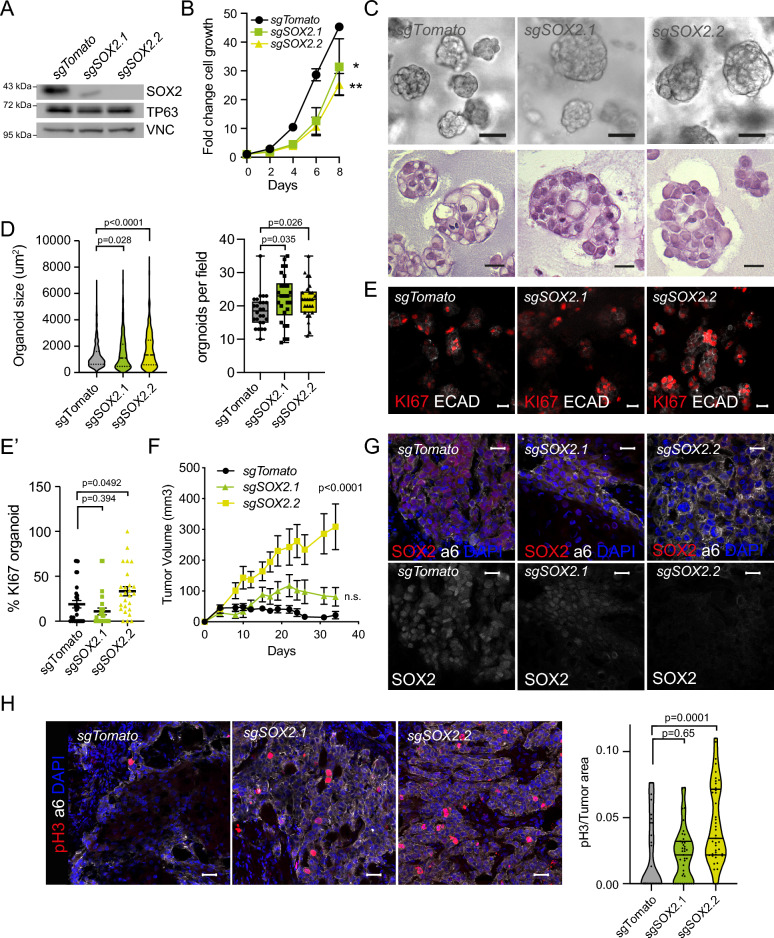
SOX2 loss drives tumor growth in SG-SCCs. (**A**) Western blot showing SOX2 protein expression in control (*sgTomato*) and SOX2 KO cells (*sgSOX2.1* and *sgSOX2.2*). (**B**) Graph illustrating the fold change growth of sgTomato and sgSOX2 cells (n = 3, Two-way ANOVA). (**C**) Brightfield (upper panel) or H&E stains micrographs of organoid sections (lower panel) illustrating the size and shape of *sgTomato* and *sgSOX2* cells after 7 days growing in BME. (**D**) Violine plot illustrating organoid size (Left) and boxplot illustrating organoids/field in *sgTomato* and *sgSOX2* A253 cells (one-way ANOVA). (**E**) Confocal micrographs of organoid sections stained with KI67 (red) in organoids grown from *sgTomato* and *sgSOX2* A253 cells. Ecadherin (ECAD, white) delimits the organoid). (**E’**) Scatter plot illustrating the percentage of KI67 positive cells per organoid (one-way ANOVA). (**F**) Tumor growth rates after intradermal injection of 100,000 sgTomato (black) and *sgSOX2* (green) human A253 cells (mean ± SEM, n = 6 transplants, two-way ANOVA). (**G**) Confocal micrographs illustrating SOX2 positivity (red) within a6 integrin positive (white) tumor cells (upper). Lower panels illustrate the remaining SOX2 expression (white) in *sgSOX2.1* tumors. (**H**) Left panel, confocal micrographs illustrating pH3 positive cells (red) within a6 integrin positive (white) tumor cells. Right panel, Violin plots representing the number of pH3 positive cells per tumor area in *sgTomato* and *sgSOX2* tumors. (one-way ANOVA). Scale bars, 50 µm.

To further test the importance of SOX2 in SG-SCC development, we mutated *SOX2* gene using CRISPR/Cas9 and two independent sgRNAs. sgRNA2 produced a complete loss of SOX2 protein, while with sgRNA1 maintained some SOX2 protein (Fig. 4A). Since SOX2 has been described to control cell proliferation, we first performed cell growth assays. Accordingly with its known role, we detected a reduction in cell growth upon *SOX2* silencing (Fig. 4B). Next, we measured the effects of SOX2 loss in self-renewal by performing organoid formation assays in ECM matrixes. Surprisingly, we observed a significant increase in the size of organoid formed by *sgSOX2* cells (Fig. 4C, D). This increase was dependent on the levels of SOX2 protein since it was more significant in *sgSOX2.2* than in *sgSOX2.1* cells. We also observed a significant increase in the number of organoids in *sgSOX2.1* cells. H&E staining of these organoids suggested that *sgSOX2* organoids contained less differentiated-like structures than *sgTomato* cells (Fig. 4C) but had similar cell density (Figure Supp. 3A). In addition, *sgSOX2.2* cells contained higher number of KI67-positive cells (Fig. 4E, E’). Unexpectedly, these data suggest that SOX2 loss enhances cancer cell self-renewal capacity in SG-SCCs.

To determine if this was also true in vivo, we injected *sgTomato* and *sgSOX2* cells intradermically into Nu/Nu mice to measure tumor growth. Both *sgSOX2*-derived tumors grew faster than control *sgTomato* tumors (Fig. 4F). Tumor growth was dependent on SOX2 levels, since *sgSOX2.1* tumors that still expressed some levels of SOX2 protein grew only slightly faster than the controls, while *sgSOX2.2* tumors (Fig. 4G), which have no detectable SOX2 protein expression, grew much faster than controls. Consistently, we quantified higher pH3 and KI67 levels in *sgSOX2.2* tumors (Fig. 4H, Figure Supp. 3B), and only a tendency in *sgSOX2.1* tumors in comparison to the *sgTomato* controls. Overall, these data demonstrate that SOX2 loss promotes tumor growth in A253-derived SG-SCCs. These results contrast with those obtained for DUSP1 mutant cells that also presented low SOX2 levels, reinforcing that the DUSP1 phenotype is not mediated by SOX2 loss.

To further understand the interconnection between DUSP1 and SOX2, we measured DUSP1 expression on SOX2 deficient cells upon U.V. irradiation (Figure Supp 3C). Interestingly, DUSP1 levels are decreased both in basal conditions and upon U.V. irradiation in *sgSOX2* cells. Reanalyzing SOX2 ChIPseq data in HNSCCs^16^, we identified that *DUSP1* is a SOX2 direct target (Figure Supp 3D), which can explain why it is downregulated in *sgSOX2* cells.

Intriguingly, when we cause DUSP1 loss as the first event, SOX2 is downregulated and there is an over-activation of JNK signaling in comparison to control cells in response to U.V. or growth in 3D conditions (Figure Supp 3C, Fig. 3F). In contrast, when SOX2 expression is lost as the first event, DUSP1 expression is decreased but pJNK levels remain similar to *sgTomato* control cells (Figure Supp 3C). This data suggests that when SOX2 is lost, DUSP1 expression may be dispensable since JNK signaling is downregulated, and in the absence of pJNK activation, cells can avoid apoptosis.

### SOX2 loss transcriptionally reprograms SG-SCCs to a more mesenchymal cell phenotype

**Figure 5:**
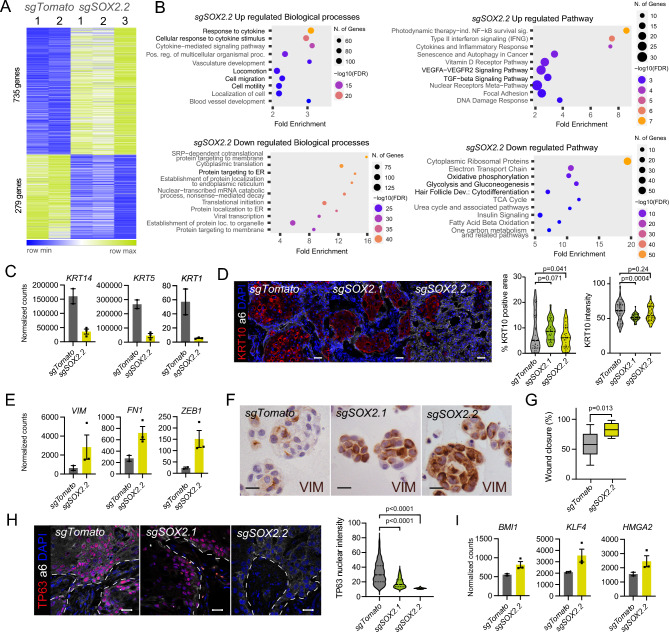
SOX2 KO SG-SCC suffer an epithelial to mesenchymal transition. (**A**) Heatmap illustration the DEG between *sgTomato* and *sgSOX2* A253 cells. (**B**) Graph showing the gene ontology biological processes (left) or pathways (right) enriched or depleted in *sgSOX2.2 cells*. In bold are highlighted the most relevant findings. (**C**) Bar graphs showing the decreased expression (Normalized counts) of squamous differentiation genes in sgSOX2.2 cells. (**D**) Left panel, Confocal micrographs illustrating KRT10 positivity (red) within a6 integrin positive (white) tumor cells. Right panel, Violin plots quantifying the intensity (right panel) or area (right panel) of KRT10 on *sgTomato* and *sgSOX2* tumors (One-way ANOVA). (**E**) Bar graphs showing the increased expression (Normalized counts) of mesenchymal genes in sgSOX2.2 cells. (**F**) Micrographs showing vimentin staining in *sgTomato* and *sgSOX2* organoids. (**G**) Box plot illustrating the % of wound closure in *sgTomato* and *sgSOX2* cells (n = 3, t-test). (**H**) Left, Confocal micrographs demonstrating a decreased TP63 expression (red) in *sgSOX2.2* grown tumors in comparison with *sgTomato* controls. a6-integrin (white) marks cancer cells; right, Violin plots representing the measurement of TP63 nuclear intensity within the a6-integrin positive cancer cells (one-way ANOVA). **I.** Bar graphs showing the increased expression (Normalized counts) of self-renewal transcription factors in sgSOX2.2 cells. Scale bars, 50 µm.

To further understand the phenotype produced by SOX2 loss, we performed RNAseq comparing control to *sgSOX2.2* cells. We rationalized that to exert a full effect we needed to have a situation with no SOX2 protein, since low levels of transcription factors (TFs) can still efficiently control gene expression. We therefore used *sgSOX2.2* cells for this analysis. SOX2 loss produced large changes in gene expression (Fig. 5A, Table Supp. 2), with 735 up-regulated and 279 down-regulated genes. Among the downregulated pathways and processes, there was a decrease in cytodifferentiation (Fig. 5B), with a decreased expression of multiple keratin genes, such as *KRT14*, *KRT5* or *KRT1* among many others (Fig. 5C). qPCR validation, confirmed these changes (Figure Supp. 3E), and staining for KRT10, identified lower positive areas with decreased expression of KRT10 (Fig. 5D). Three processes and pathways were particularly enriched in *SOX2* null cells: cell migration, vascularization, and response to cytokines (Fig. 5B). Genes of the first two pathways included TGFbeta pathway genes *TGFB2,* EMT genes such as *VIM*, *FN1*, SERPINE2, *ZEB1* or *LOXL2,* and *VEGFA*, *VEGFC* or *PDGFB* respectively (Fig. 5E) Figure Supp. 3D). We could confirm an increase of vimentin in *sgSOX2* derived organoids (Fig. 5F), and *sgSOX2* cells became more migratory in 2D scratch assays (Fig. 5G). We also observed a pronounced decrease in pathways related to protein translation and the endoplasmic reticulum, and particularly in OXPHOS, suggesting that SOX2 null cells are suffering a metabolic reprograming that could be guiding the aggressiveness of these cells. Remarkably, when we compared the DEG of *DUSP1* or *SOX2* loss of function, we find out that they control completely opposite functions (Figure Supp 3F). Keratinization genes are increased in *DUSP1*^-/+^ cells and decreased in *sgSOX2* cells, while EMT and migration genes are decreased in *DUSP1*^-/+^ cells and increased in *sgSOX2* cells. These data reinforce the idea that SOX2 and DUSP1 control opposite functions during tumors progression in SG-SCCs.

Since we and others had shown that SOX2 controls *TP63* expression^16^, and TP63 defines the expression of squamous programs in cSCCs, we analyzed the expression of TP63 in *sgSOX2* tumors. We observed a SOX2-dose dependent decrease in TP63 expression, suggesting that squamous features may be lost by a concomitant decrease in TP63 and SOX2 expression (Fig. 5H). The decrease in TP63 protein was dependent on growth conditions, since 2D cultures showed unaltered protein levels, which became undetectable when we grew *sgTomato* or *sgSOX2* cells in 3D, that better resemble tumor conditions (Figure Supp 3G).

Intrigued by this observation, we explored the expression of other stem related TFs, and we identified that the expression of *BMI1*^17^, *KLF4*^18^ and *HMGA2* was significantly increased in *sgSOX2* cells (Fig. 5I). Overall, these data suggest that *SOX2* null SG-SCC are reprogramed to a more mesenchymal phenotype and migratory capacity, with loss of TP63 and squamous markers, which could explain its more aggressive behavior.

## Discussion

Salivary gland squamous cell carcinomas are a very rare type of cancer that can originate from the basal cells of the salivary gland, having a bad prognosis in comparison to other salivary gland malignancies^8^. In this work we aimed to investigate the function of a pleiotropic protein, the protein phosphatase DUSP1, which we have shown, among others, to have pro-oncogenic or tumor suppressor function depending on the cancer type^13^.

We generated *DUSP1* *CRISPR*-KO A253 cells, which were described as well-differentiated SG-SCCs. We found that *DUSP1*^−/+^ cells had a predominant stress phenotype, caused probably by an increased JNK activity, which made these cells unable to grow 3D in ECM matrix. We observed that many of these cells suffered apoptosis in 3D, and when transplanted into mice, formed very small and disorganized tumors. Indeed, we were only able to generate heterozygous deletions of *DUSP1* gene, highlighting its essential role in this cancer type. Although heterozygous, some of the clones did not express DUSP1 protein, which could be due to the introduction of inactivating indels in WT alleles that prevented the correct DUSP1 expression. This agrees with the phenotype that we observed in lung^19,20^ and that was observed in other cancer types, where DUSP1 expression increases early during tumorigenesis and prevents JNK-mediated apoptosis. Importantly, we could recapitulate some of these findings using a DUSP1 inhibitor, highlighting the translational potential of our results.

The RNAseq analysis of *DUSP1*^−/+^ cells revealed that these cells have an increased expression of genes related to epithelial keratinization, and therefore a more differentiated phenotype. This phenotype has been shown to be linked to the loss of self-renewal capacity in cutaneous SCCs (cSCCs), through the loss of expression of the transcription factor PITX1^16^. Indeed, *DUSP1*^−/+^ cells lose the expression of SOX2 -another well-known regulator of self-renewal in cSCCs^14,21^- at the mRNA and protein level, by still undefined mechanisms. It has been shown that SOX2 is required to initiate cSCC tumors in mouse models^21^, and that SOX2 expression is required to sustain tumor growth in mouse and human orthotopic models, by regulating self-renewal^14^.

Surprisingly, when we tested the role of SOX2 inhibition, it did not recapitulate *DUSP1*^*-/*+^ phenotype or what was observed in cSCCs. Although SOX2 null cells grew slightly slower in 2D conditions, when we grew them in BME, organoids reached greater size and number than controls. Furthermore, when injected into mice, *SOX2* KO tumors grew much faster than controls. These data suggest that in the context of tumorigenesis of salivary gland basal cells, SOX2 expression might not be required to promote stemness. In fact, it could be acting as a gatekeeper of premalignant growth, and its loss could promote cancer progression. Additionally, we observed that *SOX2* KO tumors lose the expression of transcription factor TP63, which marks basal stem progenitors in the healthy salivary gland^5^. *TP63* expression has been described by us to be regulated directly by SOX2 binding to *TP63* DNA regulatory regions^16^. We demonstrated that Trp63 silencing promotes apoptosis in cSCC cells and loss of tumor forming potential. This data indicates that A253 cells could become independent of these two TFs during the progression of the disease, and the loss of this TFs promotes the loss of epithelial differentiation.

Overall, these data suggest that DUSP1 acts as an oncogene that drives tumor growth by blocking JNK activity. As for SOX2 expression in these tumors, based on these data we could hypothesize that perhaps it is maintained in earlier stages of the disease, decreasing in later stages what could lead to a decrease in epithelial differentiation. However, how SOX2 expression changes over the course of human disease, remains to be studied in cohorts of patient samples.

Analysis of SOX2 KO RNAseq threw some light into the functions that SOX2 is controlling in these cancers. While *DUSP1* KO cells have a decrease in the motility signature, with lower expression of vimentin, fibronectin and ZEB1, *SOX2* KO cells have a significant upregulation of these genes (Figure Supp 3F). This is in agreement with what is seen in cSCC, in which Sox2 and Trp63 expression characterize more epithelial tumors, while their downregulation^22^, correlated with the induction of a degree of EMT states, producing tumors with higher stemness and metastatic potential. It is also in agreement with the described function of SOX2 in oral SCC where *SOX2* silencing induced a more mesenchymal phenotype^23^. Since EMT TFs have been linked to increased self-renewal capacity, the *SOX2* KO phenotype could be induced by the expression of ZEB1, alone or in combination with other stemness TFs such as KLF4^18^, BMI1^24^ or HMGA2, which are also increased in *SOX2* KO cells and have been shown to control cancer stem cell stemness. It has been demonstrated that BMI1 functionally marks keratinocytes with cancer stem cell properties in tongue SCCs, and promotes chemotherapy and immunotherapy resistance^25,26^. The induction of EMT has been tightly linked to a metabolic switch^27^. In this regard, we observe that *SOX2* KO cells have a decreased OXPHOS signature. Exploring the scRNAseq datasets of mouse SG-SCC -induced by Wnt activation and BMP inhibition- we realized that *Dusp1* and *Sox2* are upregulated in a specific CSC cell cluster (CSC2)^9^. Importantly, this cluster of cells is also enriched in OXPHOS genes. We hypothesize that these genes are controlled directly by SOX2 expression, since our KO cells are losing the expression of key components of the pathway. Interestingly, we also observed that some of the signature genes enriched in the next population on the trajectory of cancer cells (luminal cells) are upregulated in SOX2 KO cells, suggesting than SOX2 expression would need to decrease to allow the transition.

One of the most striking observations that we made is that SOX2 or DUSP1 loss produce different phenotypes depending on the order of the events. When DUSP1 expression is lost first, although SOX2 expression decreased almost to undetectable levels, the cell death promoted by the activation of JNK seems to be the dominant phenotype. Oppositely, when SOX2 expression is lost first, JNK signaling seems to be impaired regardless of the decrease in DUSP1 expression, its negative regulator, and a pro-tumoral phenotype is predominant. This data perhaps indicates that DUSP1 is required early during carcinogenesis to prevent JNK activation, but once SOX2 expression declines through the progression of the disease, it becomes expendable. How SOX2 RNA decreases upon DUSP1 loss and how JNK signaling is constrained in SOX2 absence remains to be studied. One possibility is that the decrease of SOX2 expression is due to off-target effects of DUSP1 sgRNAs and the constitutive CAS9 used to engineer our cell lines.

In summary, our work demonstrates the role of two relevant genes (DUSP1 and SOX2), in a rare type of cancer such as SG-SCC. DUSP1 acts as an oncogene since its expression is required to constrain JNK signaling and prevent apoptosis. SOX2 on the other hand, has an unexpected role since the loss of its expression enhances both self-renewal and EMT, two well-known processes that drive cancer progression. Overall, these data suggests that SOX2 expression could be a predictor of good prognosis in SG-SCCs. Although these results seem promising, further analyses need to be done in order to explore the expression levels of SOX2 and DUSP1 in patient samples and calculate their correlation with the progression of the disease.

## Methods

### Mice

6-week-old, female Nude (NU/NU [088] Charles River) mice were used for orthotopic transplantations and xenograft studies. Tumors were detected by palpation, measured with digital calipers to calculate tumor volumes (V_Tumor_ = (π/6) x l x w^2^, where l = length in mm and w = width in mm). All animal experiments were performed in accordance with the guidelines and approval of the Institutional Animal Care and Use Committee at *Instituto Investigaciones Biomédicas Alberto Sols*. All authors complied with the ARRIVE (Animal Research: Reporting of In Vivo Experiments) guidelines. All mice were grown under circadian light (12 h/12 h) at (22 ± 2) °C with freely available clean water and feed.

### Cell lines

Human A253 (ATCC) cell line was grown in DMEM supplemented with 10% fetal bovine serum (FBS), L-glutamine (Invitrogen) and Pen/Strep solution.

For stable cell line generation, VSV-G pseudotyped lentivirus was produced by PEI transfection of 293 T cells (ATCC) with pLKO *sgRNA*-carrying vectors and helper plasmids pMD2-VSVg and pPAX2 (Addgene plasmid 12259 and 12260, respectively). 293 T cells were maintained in DMEM (Gibco) supplemented with 10% FBS and Pen/Strep solution. Viral supernatant was collected 48 and 72 h after transfection and filtered through 0.45-μm polyvinylidene difluoride filters. For infections, 3 × 10^5^ cells were plated in a single well of a 6-well plate, incubated with a 1:2 dilution of viral supernatant containing 8 μg/mL Polybrene. Forty-eight hours after infection, puromycin-resistant cells were selected with 1 μg/mL puromycin (Sigma-Aldrich).

Growth curves of cultured cells were measured by crystal violet staining and reported as p < 0.05 calculated by Student’s t test. Cells were exposed to 20 J/m^2^ of U.V.C and protein collected at the indicated time points after exposure. A253 control and DUSP1KO or sgSOX2 cells (1 × 10^5^ cells/injection) were prepared in 50% Matrigel and injected intradermally in Nude recipient mice.

### CRISPR/Cas9 knockouts in SCC cells

The *DUSP1* exon2 was deleted with two *sgRNAs* targeting the 5´and 3´ intronic regions of the gene. SOX2 was targeted with two independent sgRNAs guided to the begging of SOX2 exon. The most efficient guide RNAs were predicted using the CRISPR design tool from Benchling. *sgRNA* against dTomato was used as a control. *sgRNA* sequences were cloned into pLKO U6-puro vector (Addgene 52963). After *sgRNA* transduction and selection, cells were subsequently infected with lentiCas9-Blast (Addgene 52962) and selected with Blasticidin for 3 days (5 μg/ml). Single cell clones were selected and screened by PCR of genomic DNA. Clones were further validated by Western blot analysis with anti-DUSP1 antibodies. *sgRNA* sequences are listed in Table Supp. 3.

### Scratch assay

500,000 cells were seeded in 6-well plates. Cells were treated with 8 ug/ml of mitomycin for 2 h. Subsequently, wounds were created using a 200ul pipette tip, washed with 1 × PBS, and incubated in fresh medium. Images were taken at 0, 16, 24 and 48 h until scratch closed. ImageJ was used to quantify the area of each scratch, and the percentage of closure was normalized to time 0.

### Organoid 3D culture

50.000 cells of control or CRISPR engineered cells were resuspended in 70% BME in Advanced media (Gibco, GlutaMAX 1x, P/S 1 × and HEPES 10 mM), plated in low adherence plates in 50ul drops^28^, and dried upside down until the BME had solidified. Organoids were covered and grown in Advaced Supplemented Medium (Advanced medium supplemented with B-27 1X, Nicotinamide 10 mM, N-Acetylcysteine 1.25 mM, EGF 50 ng/ml, FGF-2 5 ng/ml, FGF-10 10 ng/ml, CHIR 3uM, Y-27632 10uM, Noggin 100 ng/ml, R-spondin 200 ng/ml, and Forskolin 1 uM, PGE2 1uM and A83-01 500 nM). Media was replaced every other day; organoids were grown for up to 10 days. Bright field images were taken every day to quantify the number and size or organoids using FIJI. Full drops were fixed with 4% PFA 20 min, and later paraffinized using standard protocols.

### Apoptosis and cell cycle assays on organoids

Organoids were retrieved by dissolving BME with Dispase II (Roche) (100 mg/ml stock) diluted 1:100 in ADV +  +  + medium. Rock inhibitor was also added to a final concentration of 10 uM. Organoids were incubated at 37 °C for 30 min and retrieved in a 15 ml tube with equal volume ADV+++ and a p1000 tip cut at the end. ADV medium was added until 10 ml, and tubes containing the organoids were incubated on ice for at least 20 min. Organoids were centrifuged at 300 g 5 min at 4 °C, supernatant was removed, and pellet was once again resuspended with up to 10 ml ADV medium, and left on ice another additional 20 min. After centrifugation, 1 mL TrypleExpress was added cells incubated at 37 °C for 5 min to digest organoids into single cell suspension. 10% FBS was added to stop digestion and centrifuged. Supernatant was removed and pellet was washed with 1X PBS. For cell cycle, cells were incubated for 45 min at 37 ºC with 10ug/ml of Hoechst. For apoptosis assay, cells were incubated with Annexin V and 7ADD (Miltenyi) for 15 min at RT. Cells were analyzed in a FACS Celesta and data analyzed in FlowJo software.

### Immuno-fluorescence and imaging

Unfixed tumors were embedded in OCT (Tissue Tek). Frozen sections were cut to a thickness of 10 μm on a Leica cryostat and mounted on SuperFrost Plus slides (Fisher). Slides were air-dried for 10 min, then fixed for 10 min with 4% formaldehyde, rinsed with PBS, permeabilized with 0.5% Triton X-100 in PBS for 15 min, then blocked for 1 h (5% normal donkey serum, 1% BSA, 0.3% Triton X-100 in PBS) and incubated with primary antibody diluted in blocking buffer at 4 °C overnight. After washing with PBS, secondary antibodies, conjugated to Alexa 488, RRX or Alexa 568, and DAPI (83218, AnaSpec) were diluted in blocking buffer and incubated with the slides for 1 h at room temperature (RT). After washing, slides were mounted in ProLong Gold (Invitrogen). Imaging was performed using a Leica TCS SPE Confocal Microscope or Zeiss Cell Observer. Images have been analyzed in ImageJ or Cell Profiler. Cell density was measured as the number of nuclei (DAPI) on the area stained by integrin a6. Proliferation rates or cell death was determined as the number of pH3, KI67 or Casp3 positive cells on the area stained by integrin a6. Antibodies for immunofluorescence were CD49f. (1:200; Biolegend, 313618), SOX2 (1:1000; Abcam, Ab92494), Ki67 (1:1000; Epredia, RM9106-50), TP63 (1:1000; Cell Signaling, 13109), pH3 (1:400; Cell Signaling; 9701), Caspase 3 (1:1000; Cell Signaling; 9664), KRT10 (Biolegend; 1:400; 905401).

### Immunohistochemistry

3 µm paraffin sections from control or CRISPR engineered organoids were dewaxed and hydrated (2 × xylene, 2 × 100% ethyl alcohol, 2 × 95% ethyl alcohol, 1 × 70% ethyl alcohol and 1 × 50% ethyl alcohol for 3 min each). After rinsing in PBS, slides were placed into a PTLink (DAKO) (98 °C for 20 min) in EnVision Flex Target Retrieval Solution High pH (DAKO) for heat-induced antigen retrieval. Endogenous peroxidase activity was blocked using EnVision FLEX Peroxidase-Blocking Reagent (DAKO) for 10 min at RT after wash in EnVision FLEX wash buffer (DAKO). Following washing, slides were incubated with primary antibodies diluted in EnVision FLEX antibody diluent (DAKO) and slides incubated Over/Night at 4C (Vimentin (1:1900, abcam, 92,547), Caspase3 (1:1000, Cell signaling, 9662)). Slides were washed with EnVision FLEX wash buffer (DAKO) and incubated with EnVision FLEX + mouse (DAKO) for 30 min at RT. After final washes, staining was developed by incubating with EnVision FLEX DAB + Chromogen (DAKO) for 5 min at RT. Slides were counterstain with hematoxylin, dehydrated (1 × 50% ethyl alcohol, 1 × 70% ethyl alcohol, 1 × 95% ethyl alcohol, 2 × 100% ethyl alcohol and 2 × xylene for 3 min each) and mounted with Permount (SP-15–100, Fisher Scientific).

### Western blotting

Cell lysates from culture cells were prepared using RIPA buffer (150 mM sodium chloride, 0.1% Triton-X 100, 0.5% SDS and 50 mM Tris pH 8 in ddH2O) with complete Mini EDTA-free protease inhibitor tablets (04693159001, Roche). Protein concentrations were determined using the Bradford protein Kit (5000001, BioRad) following the instructions. Lysates were boiled with Laemmli buffer (5×: 6% SDS, 15% β-mercaptoethanol, 30% glycerol, 0.006% bromophenol blue, 0.188 M Tris–HCl) for 10 min at 95 °C. Protein ladder used was EZ-Run Molecular Weight Markers (10638393, Fisher). 30 μg of protein was loaded per lane. Gel electrophoresis was performed using a 10 or 12% Bis–Tris-gel run for 75–150 min at 120 V, gel was transferred for 1.5 h at 4 °C at 100 V to a 0.45 μm nitrocellulose membrane (Whatmann) and transfer was assessed by Ponceau S staining (0.1% (w/v) Ponceau S in 5% (v/v) acetic acid). Membranes were sectioned in pieces to incubate them simultaneously with different antibodies, then were blocked with 5% non-fat dry milk in TBST, then incubated with primary antibodies diluted in blocking overnight at 4 °C with gentle agitation. Membranes were rinsed with TBST before incubating with horseradish peroxidase-conjugated secondary antibodies diluted in blocking buffer for 1 h at RT. Membranes were washed with TBST before incubating them with SuperSignal™ West Pico (Life Technologies #34080) and exposed in a UVITEC Chemidoc. Antibodies used for western blotting were DUSP1 (1:1,000; Cell Signaling, 48625), pERK (1:1,000; Cell Signaling, 9101), ERK (1:2,000; Santa Cruz, sc154G), pJNK (1:1,000; Cell Signaling, 4668), JNK1, (1:1,000; Santa Cruz, 474), Vinculin (1:10000; Santa Cruz, 73614), SOX2 (1:1000; Abcam, Ab92494), HRP donkey anti-rabbit IgG (1:10,000; Invitrogen, A27036), HRP donkey anti-mouse IgG (1:2000; Santa Cruz, 516102), HRP donkey anti-goat IgG (1:1,000; Merck, P107P). Intensity was quantified using FIJI in at least 3 independent experiments.

### Quantitative reverse-transcription PCR

mRNA was isolated using Qiazol (Qiagen) and Direct-zol RNA Mini Prep Kits (R2052, Zymo Research). Samples were quantified using a Nanodrop spectrophotometer (Thermo Scientific). Complementary DNA was synthesized from 1.5 μg of total RNA using NZY First-Strand cDNA Synthesis Kit with random primers (NZYTech). qRT–PCR was performed with KAPA SYBR® FAST (Rox) (KK4602, Kapa) on a StepOnePlus™ Real-Time PCR System (Applied Biosystems). Measurements were recorded in triplicate. Differences between samples and controls were calculated based on the 2^−ΔΔCT^ method and normalized to RPLP0. For detailed list of primer sequences, see Table Supp 3.

### RNA-seq library preparation

Total RNA was extracted from 2 × 10^5^ control or CRISPR engineered cells using the RNA Microprep Kit (Zymo) according to manufacturer’s instructions. RNA quality was defined using an Agilent 2100 Bioanalyzer before we prepared Poly(A) + selected, multiplexed, paired end libraries with the Illumina TruSeq RNA preparation kit. Multiplexed libraries have been sequenced on an Illumina NovaSep6000 Genome Analyzer using the 150-base pair paired end read method.

### RNA-seq analysis

Human hg38 assembly version was used for the RNA-sequencing alignment, transcriptome quantification and differential expression analysis. More specifically, HISAT2 was used for aligning sequenced reads. Transcriptome quantification and differential expression analysis was performed using the HTseq protocol^29^ and DESeq2^30^. Differential gene expression analyses were performed between *Dusp1*^+/+^ *Dusp1*^−/+^ clones in replicates*,* and between *sgTomato* and *sgSOX2.2* in two independent KO experiments. Area proportional Venn diagrams were generated with BioVenn (Hulsen et al., 2008). Gene ontology analyses were performed with the Database for Annotation, Visualization and Integrated Discovery (DAVID) version 6.7^31^ and ShinyGO. Heatmaps were done using Morpheus (https://software.broadinstitute.org/morpheus).

### Statistics

All experiments were carried out single blinded. All sgRNA-mediated knockdown experiments in vivo and in vitro were repeated three independent times with biological replicates. All quantitative data were collected from experiments performed at least in triplicate, and expressed as mean ± s.d., 95% confidence interval, min/max or s.e.m. Differences between groups were assayed using unpaired or paired two-tailed Student’s t-test, or Mann–Whitney test using Prism 10 (GraphPad Software). Box-and-whisker and violin plots are used to describe the entire population without assumptions about the statistical distribution. Significant differences were considered when P < 0.05. The intensity of TP63 positive cells was measured in at least 5 fields per condition using FIJI and plotted in Prism 10. Western blots were quantified using FIJI. All other graphs were prepared in Prism 10. Figures were prepared using Adobe Photoshop and Illustrator 2024.

### Supplementary Information

Supplementary Figures.

Supplementary Information 1.

Supplementary Information 2.

Supplementary Information 3.

## Data Availability

The datasets generated during the current study are available in the GEO repository (GSE253468).
