# Supplementary material for: DUSP1 and SOX2 expression determine squamous cell carcinoma of the salivary gland progression
Source: Sci Rep. 2024 Jul 1;14:15007. doi: 10.1038/s41598-024-65945-x (PMC11217270; doi:10.1038/s41598-024-65945-x)

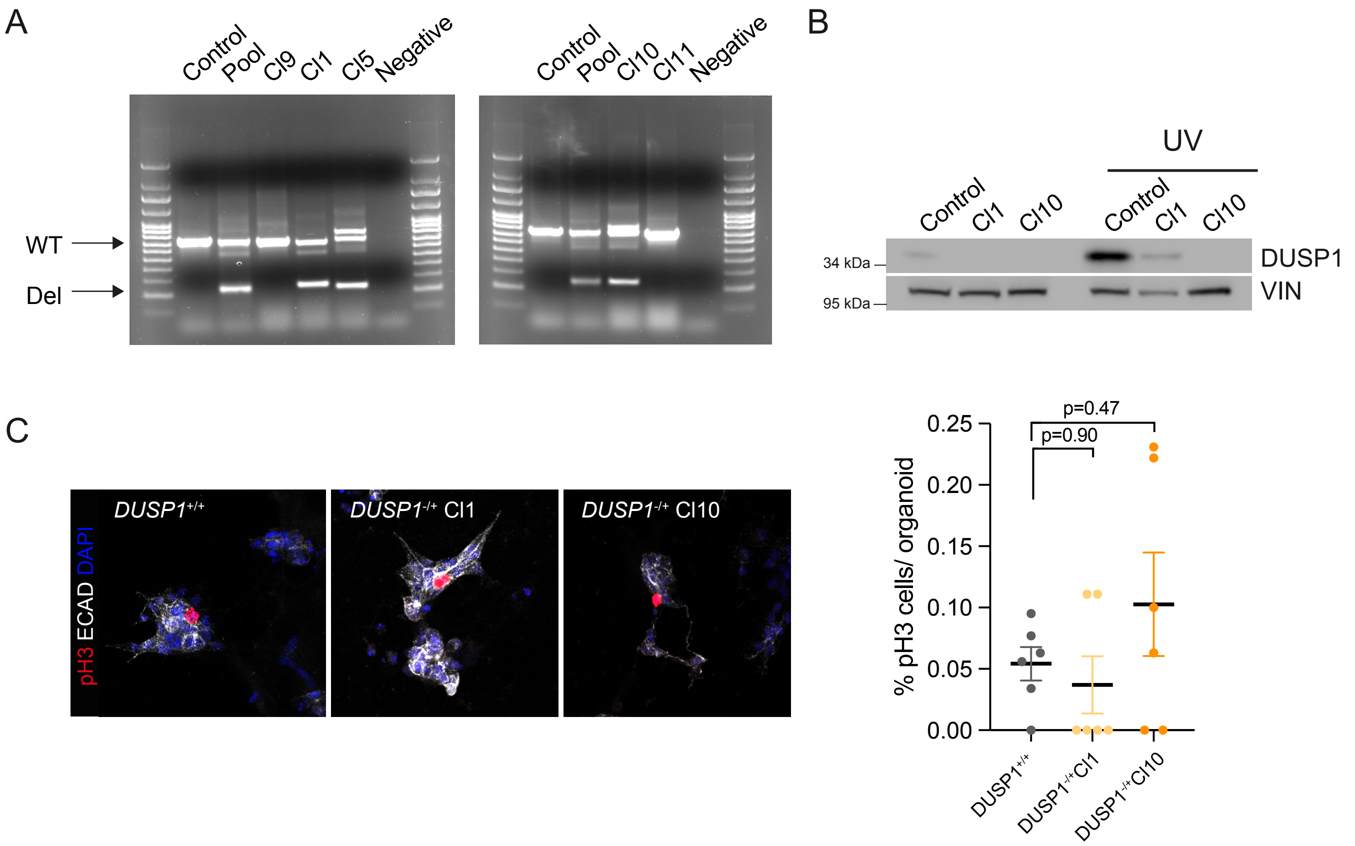

Figure Supp. 1. A. Genotyping confirming heterozygus deletion of DUSP1 in different clones. **B.** Western blot illustrating DUSP1 expression after its stimulation with U.V. radiation in DUSP1$^{+/+}$ and DUSP1$^{-/+}$ cells. **C.** Letf panel, confocal micrographs illustrationg pH3 positive cells (red) in DUSP1$^{+/+}$ and DUSP1$^{-/+}$ organoids. Ecadherin (ECAD, white) demarks the organoid. Right panel, Scatter plot showing the percentaje of pH3 positive cells per organoid (one-way ANOVA).

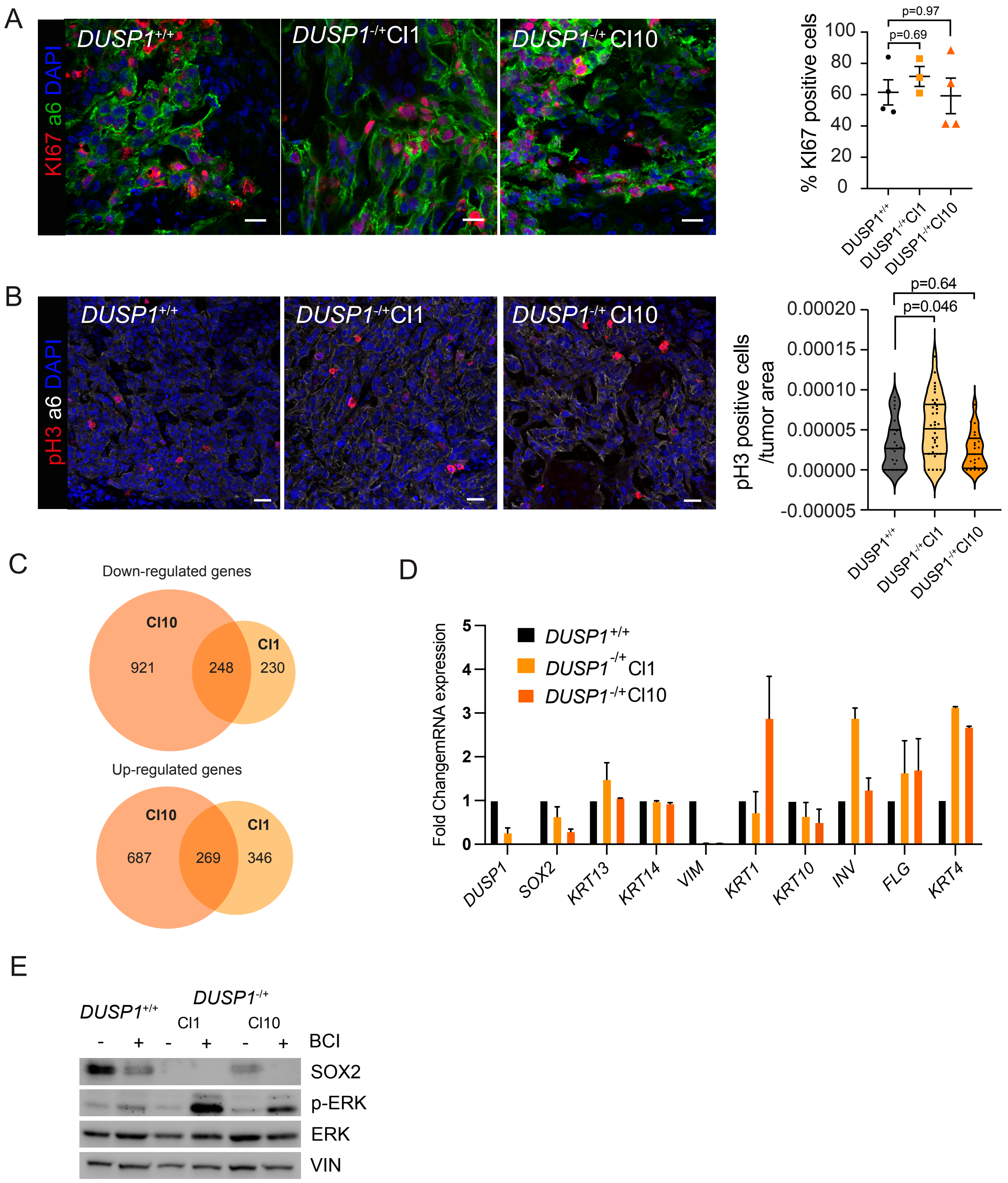

Figure Supp. 2. A. Left panels, Confocal micrographs illustrating KI67 positivity (red) within a6 integrin positive (green) tumor cells. Right panel, Graph illustrating the percentage of KI67 positive cells (one-way ANOVA). **B.** Right panel, Confocal micrographs illustrating pH3 positivity (red) within a6 integrin positive (white) tumor cells. Left panel, violin plots quantifying the number of pH3 positive cells per tumor frame (one-way ANOVA). **C.** Venn diagrams illustrating the differential expressed genes commonly downregulates (upper) or upregulated (lower) in both DUSP1$^{-/+}$ clones in comparison to control cells (DUSP1$^{+/+}$). **D**. Fold change mRNA expression of epithelial and mesenchymal genes in DUSP1+/+ and DUSP1-/+ clones. **E.** Western blot illustrating the decrease in SOX2 expression after the treatment with the DUSP1 inhibitor BCI (5uM) for 24 hours. Scale bars, 50μm.

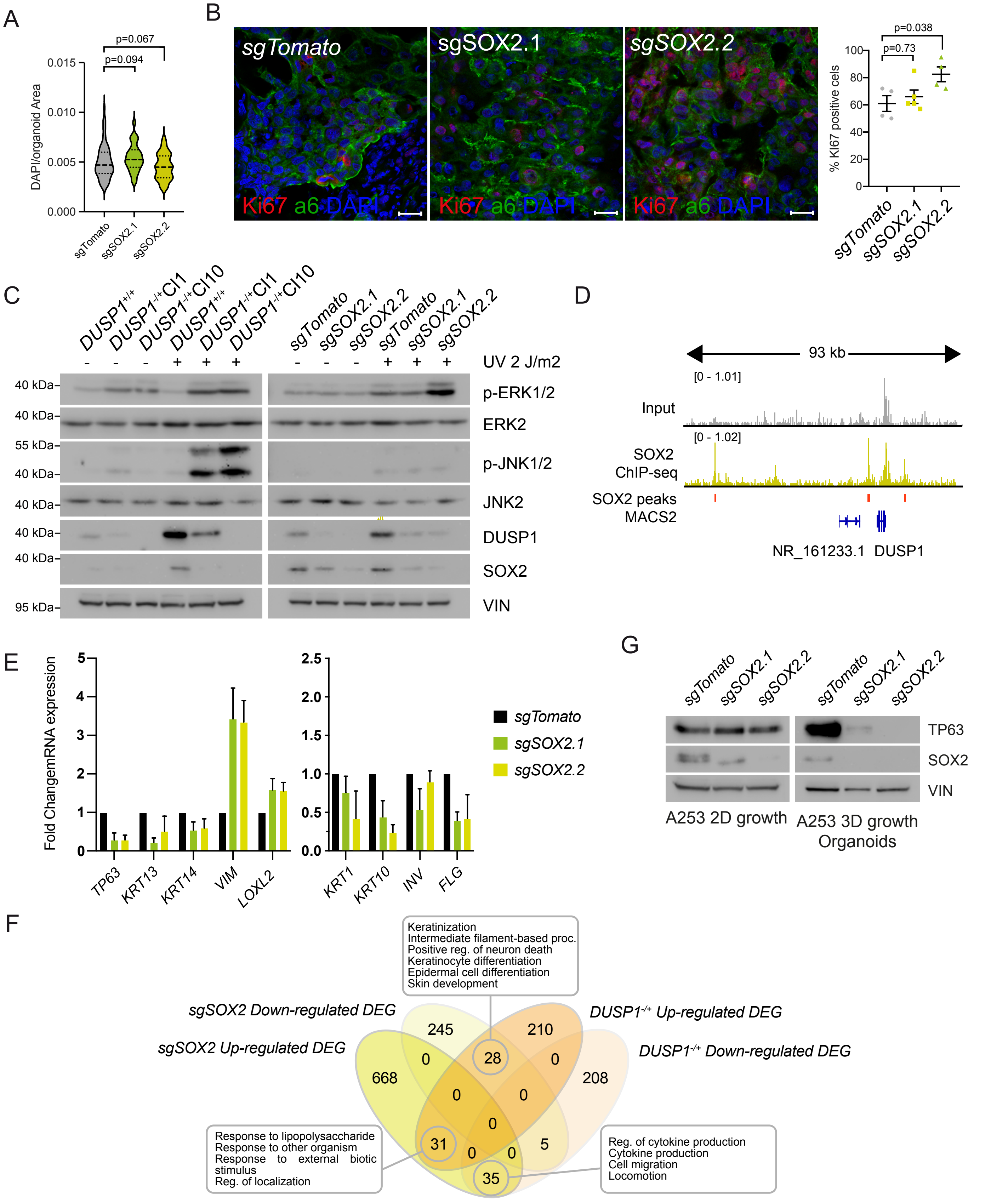

Figure Supp. 3. A. Violin plot illustrating organoid density as the number of DAPIs per organoid area (one-way ANOVA). **B.** Letf panels, Confocal micrographs showing Ki67 staining within a6 integrin positive (green) tumor cells in *sgTomato* and *sgSOX2* tumors. Right panel, Graph illustrating the percentage of KI67 positive cells (one-way ANOVA). **C.** Western blot illustrating SOX2 and DUSP1 expression in *DUSP1*$^{-/+}$ and control cells and *sgSOX2* and *sgTomato* cells 1 hour after U.V. irradiation. In the same western blot, the expression of pERK or JNK is evaluated. **D.** SOX2 and input ChIP-seq tracks on DUSP1 locus. SOX2 peaks MACS boxes identify the significant enrichment of SOX2 ChIP signal over the input. E. Fold change mRNA expression of epithelial and mesenchymal genes in *sgTomato* and *sgSOX2* cells. **G.** Western blot illustrating SOX2 and TP63 expression on *sgTomato* and *sgSOX2* cells grown in 2D regular conditions or 3D on BME as organoids. F Venn diagram illustrating the common up or down regulated DEGs on *DUSP1*$^{-/+}$ or *sgSOX2* cells vs. their controls. Grey boxes describe the GO biological processed enriched in the indicated set of genes. Scale bars, 50µm.

Figure 1A

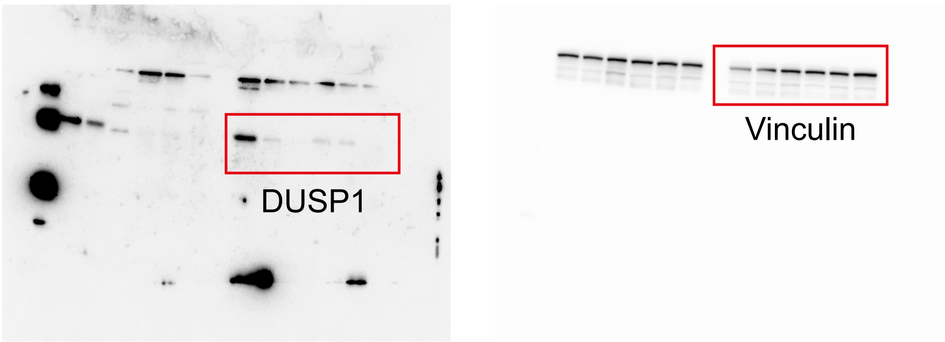

Figure 1B

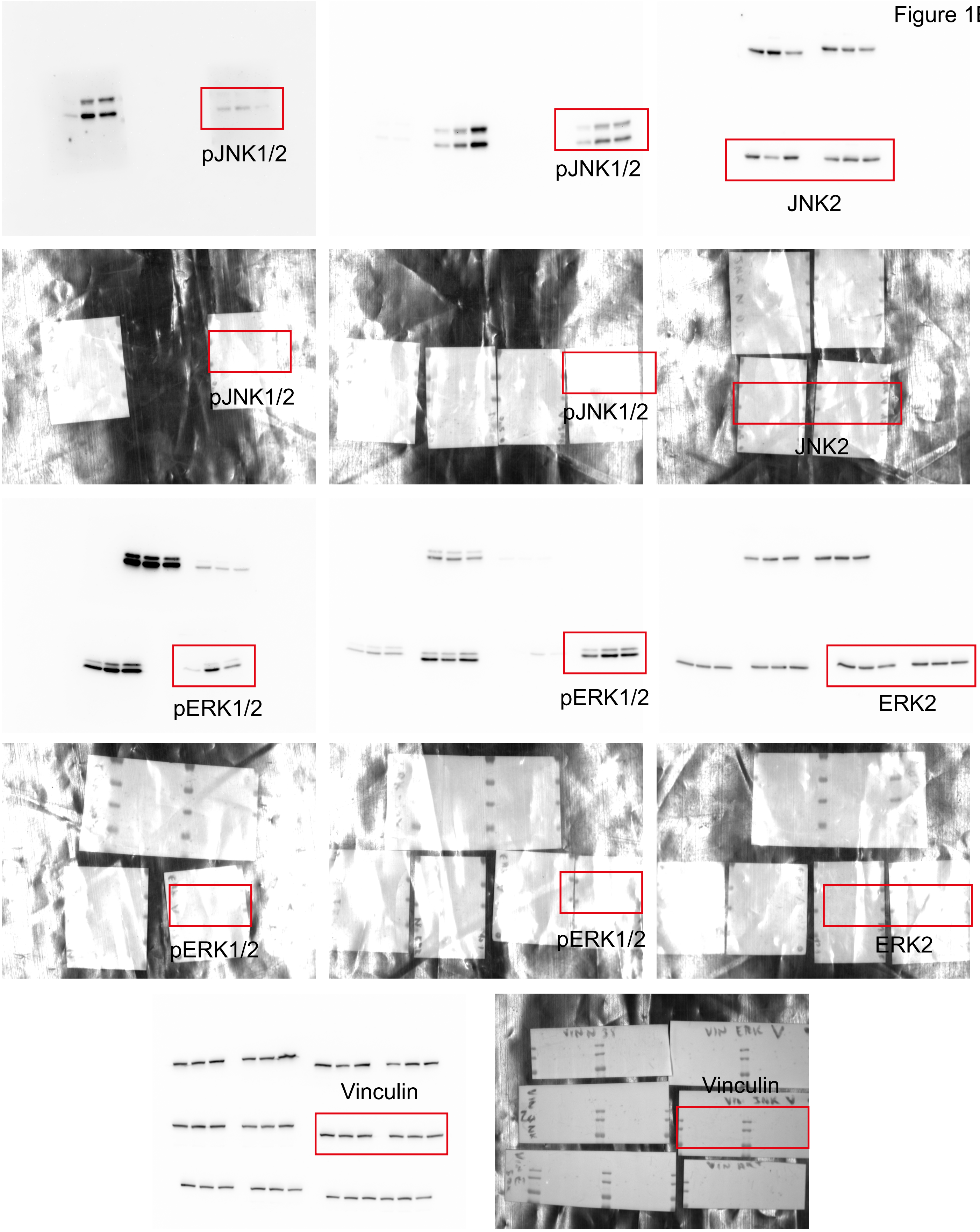

Figure 1I

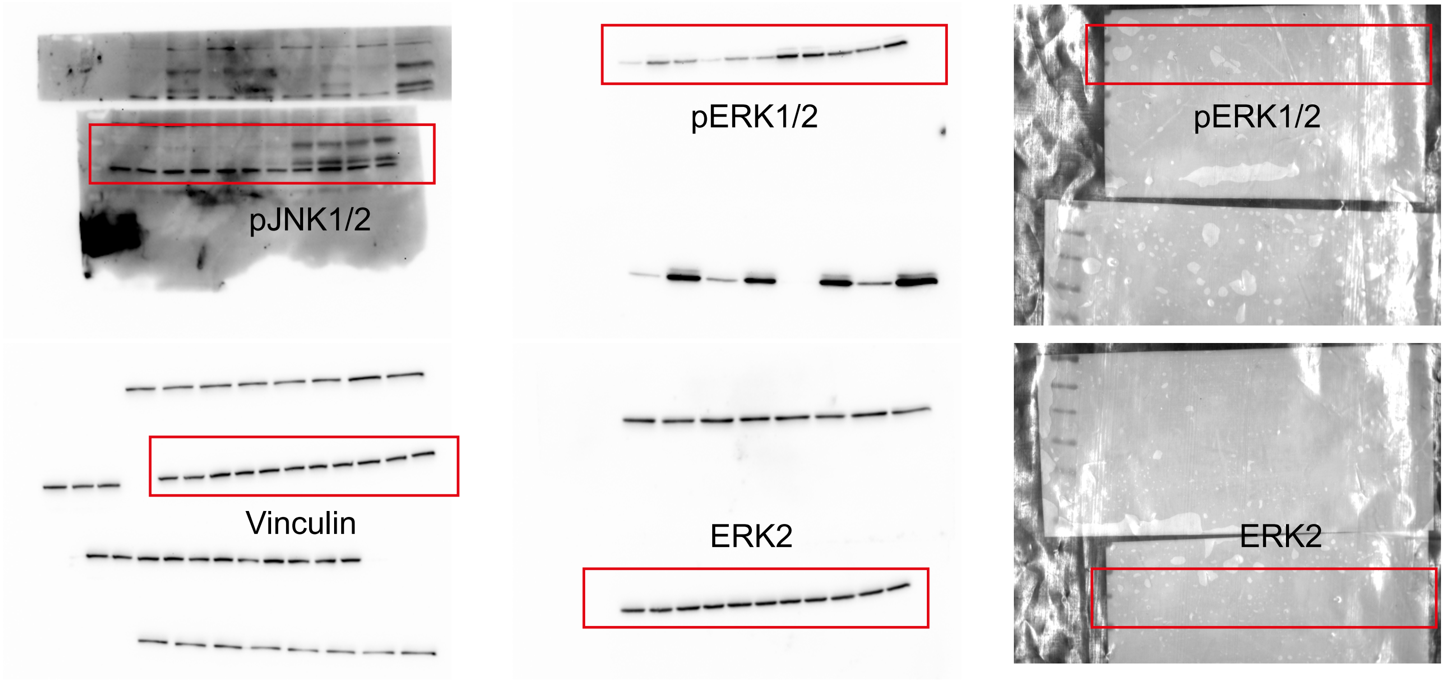

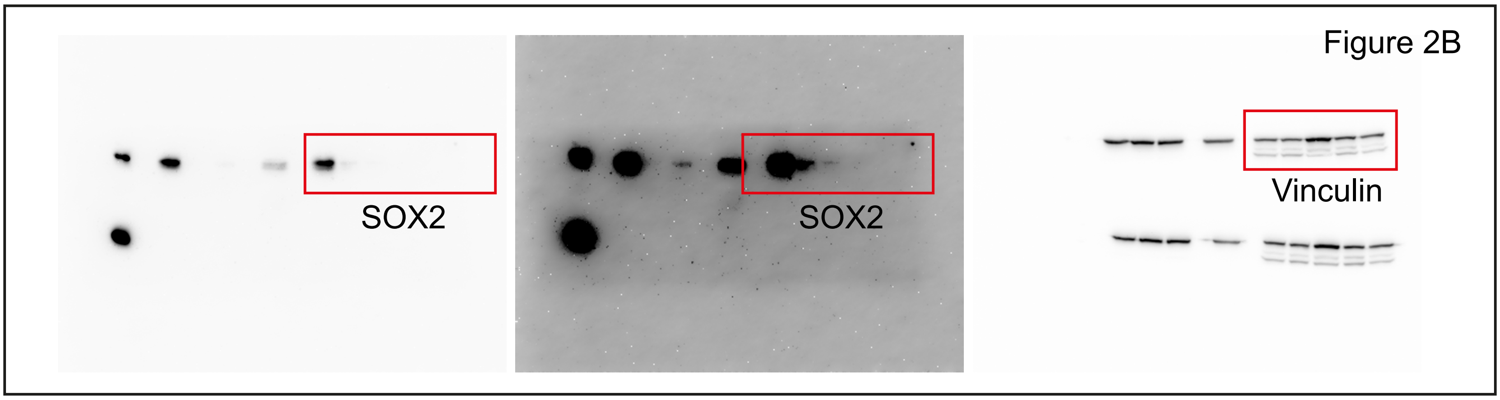
Figure 2B
SOX2
SOX2
Vinculin

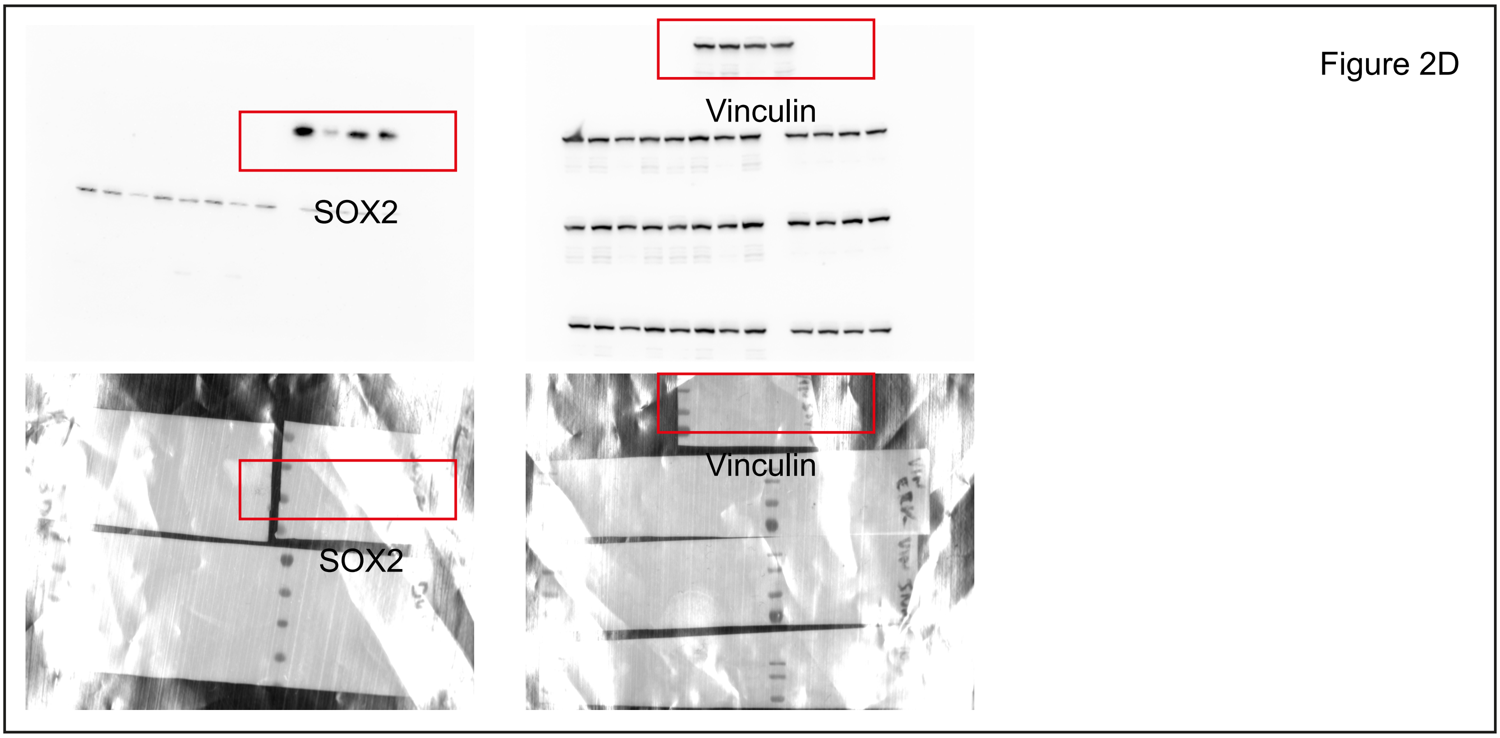
Figure 2D
SOX2
Vinculin
SOX2
Vinculin

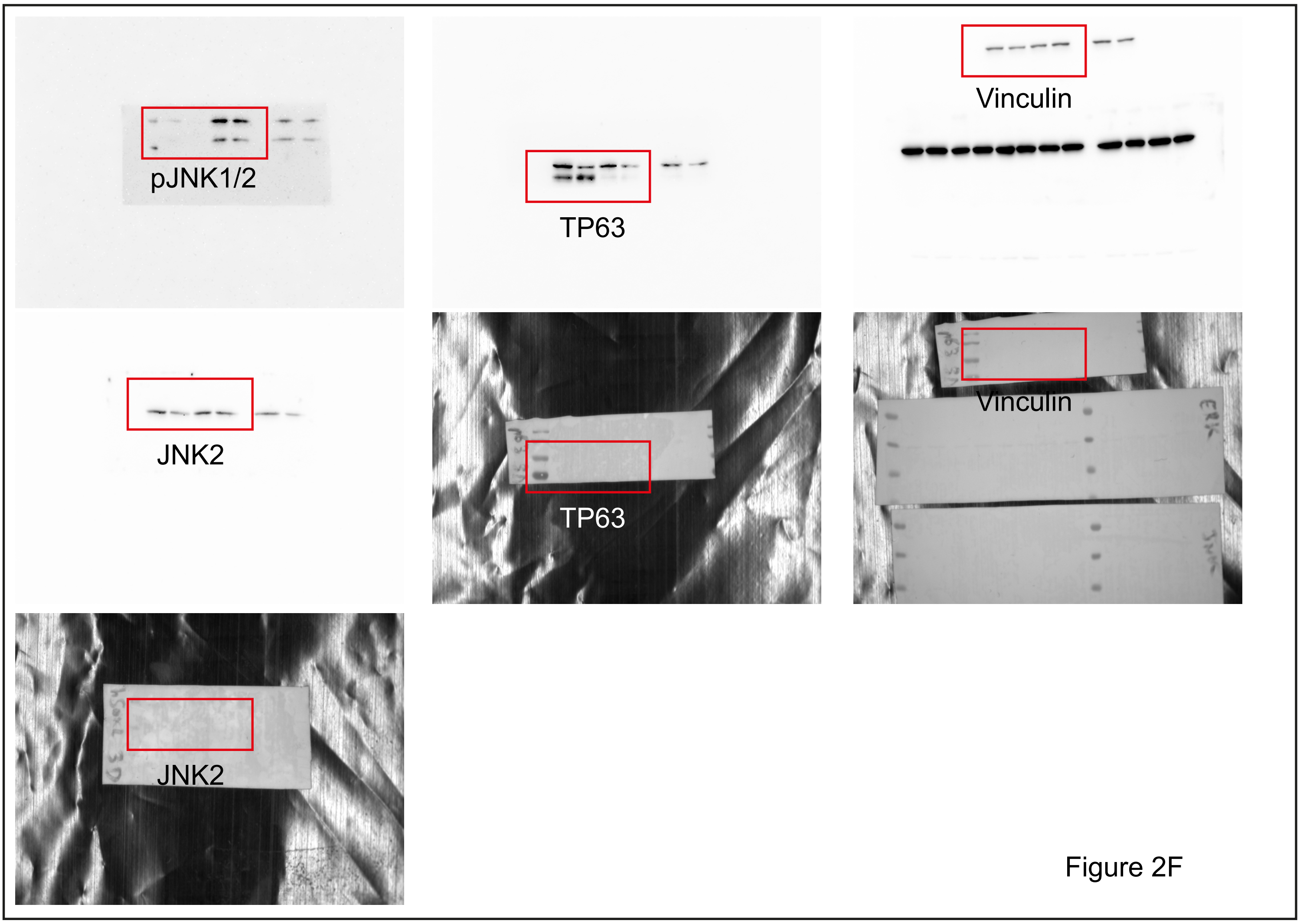
pJNK1/2
TP63
Vinculin
JNK2
TP63
Vinculin
JNK2
Figure 2F

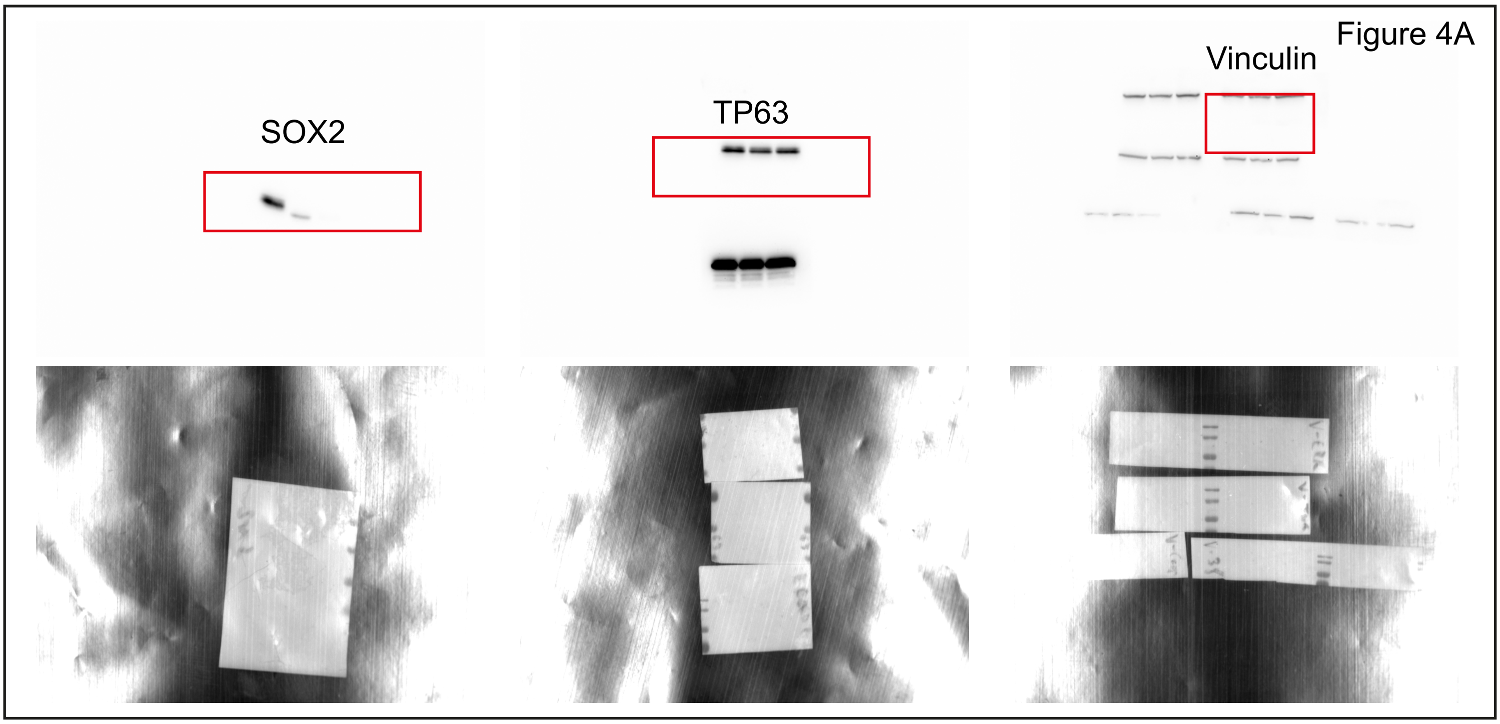
Figure 4A
SOX2
TP63
Vinculin

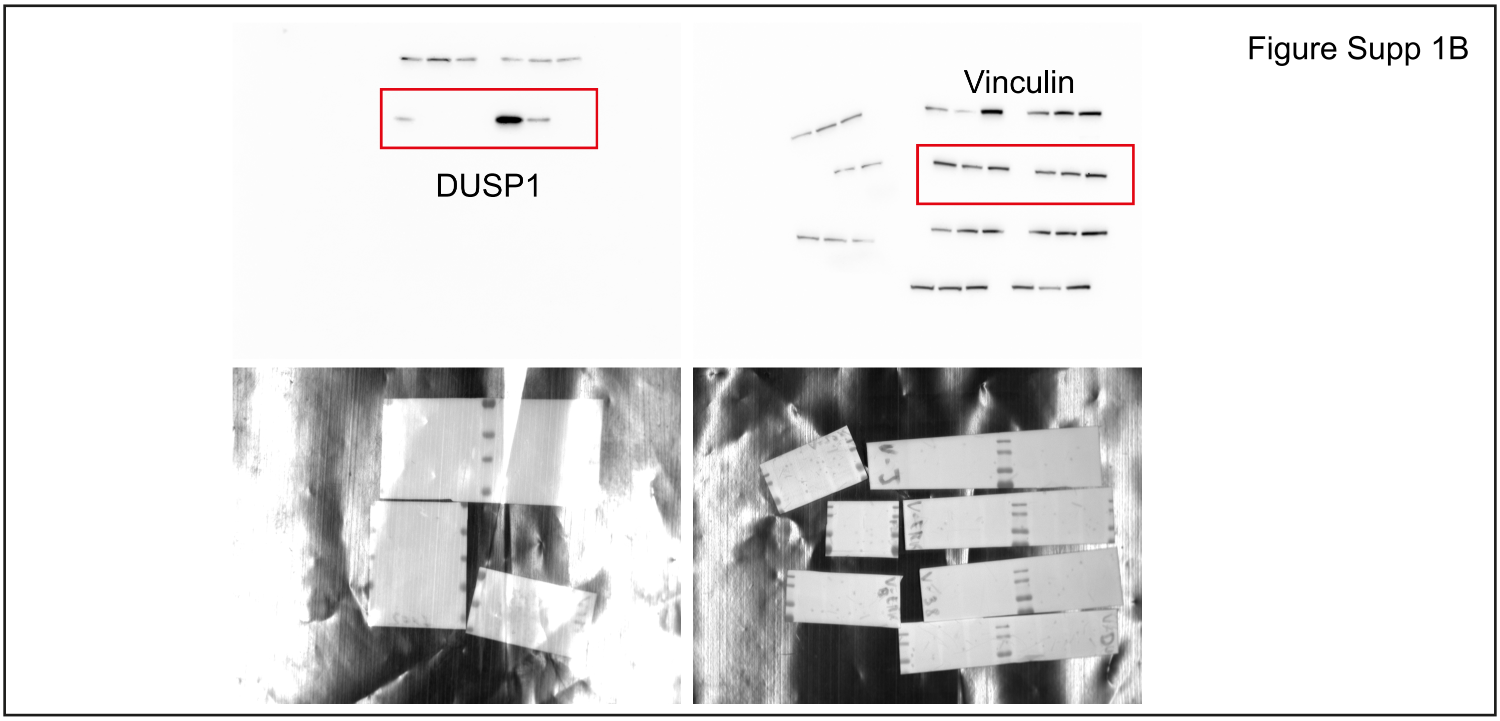
Figure Supp 1B
DUSP1
Vinculin

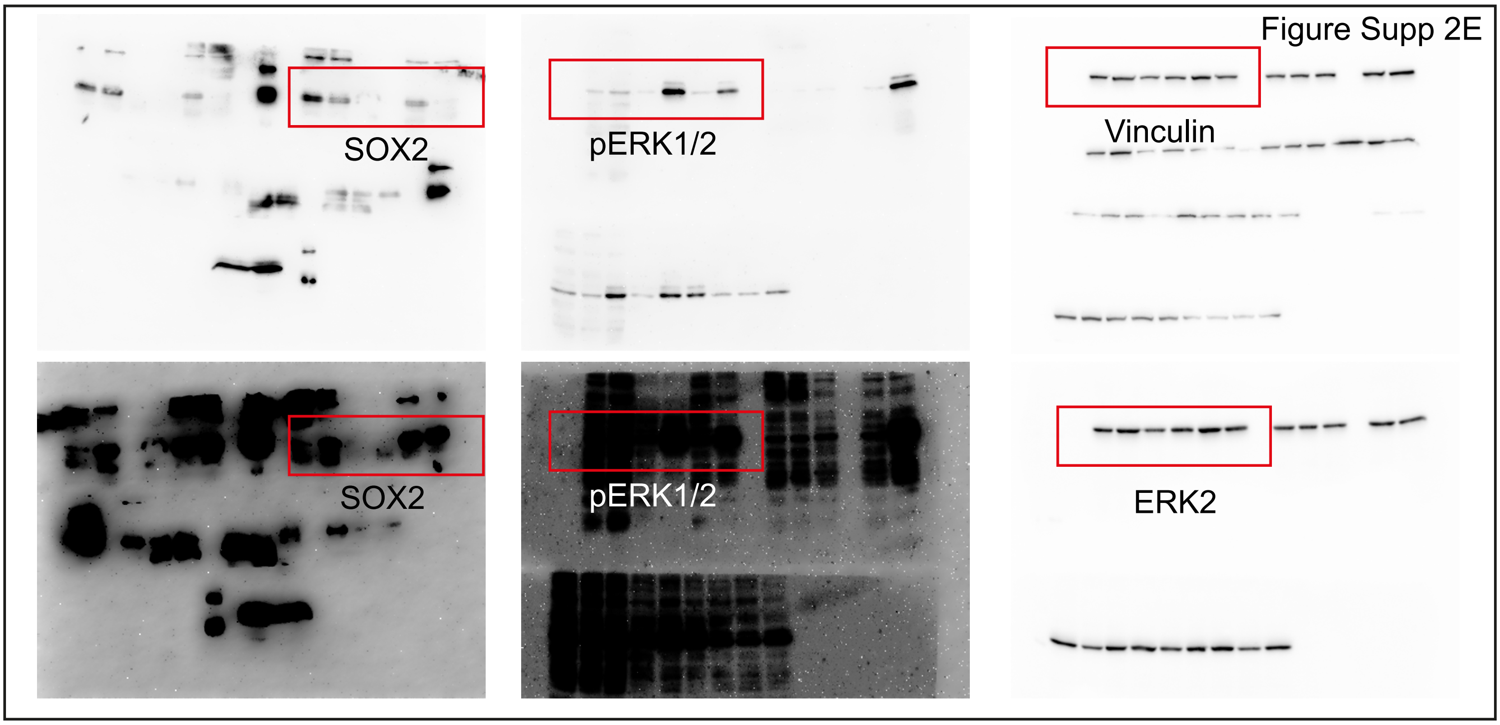
Figure Supp 2E
SOX2
pERK1/2
Vinculin
SOX2
pERK1/2
ERK2

Figure Supp 3C

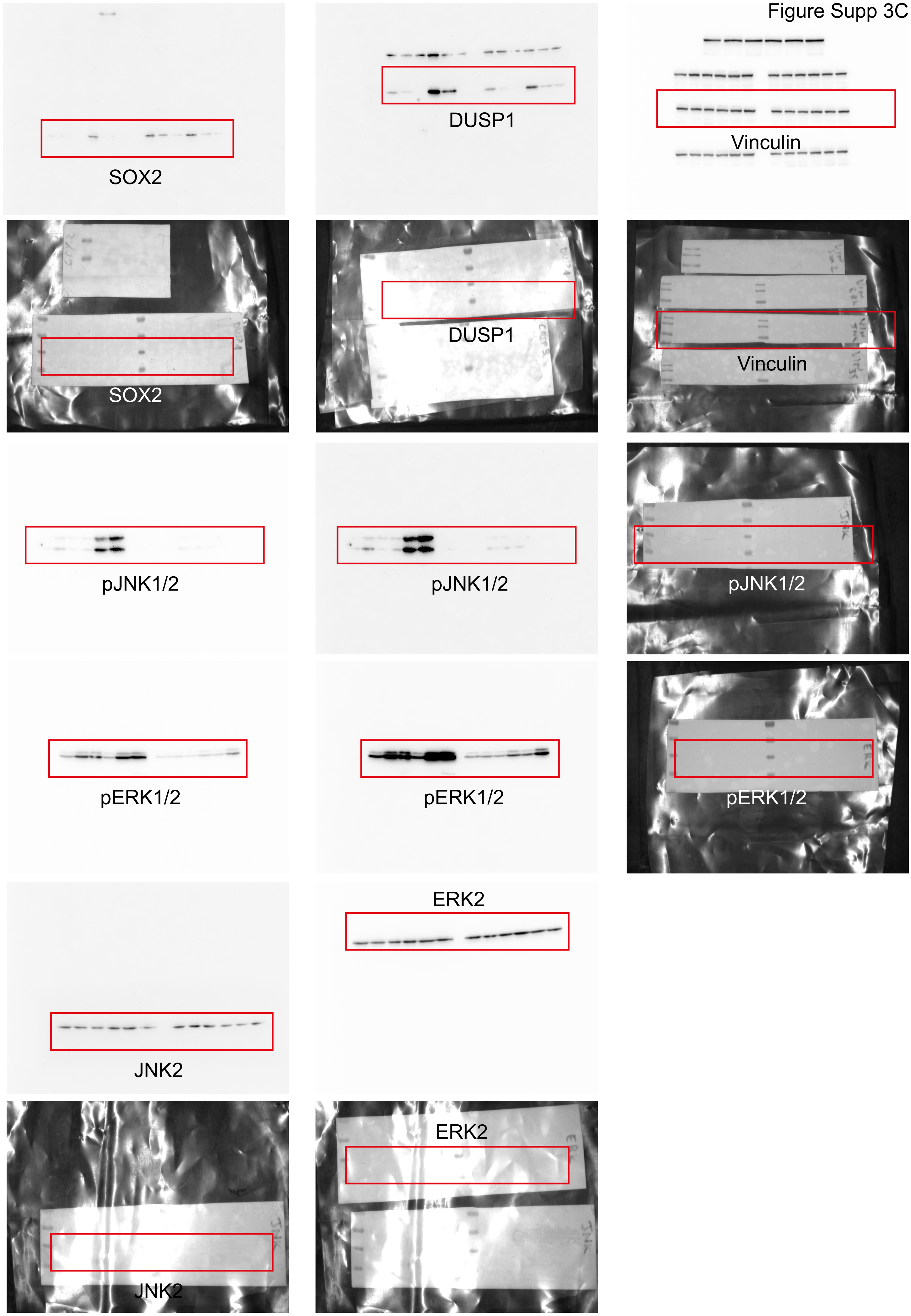

Figure Supp 3G

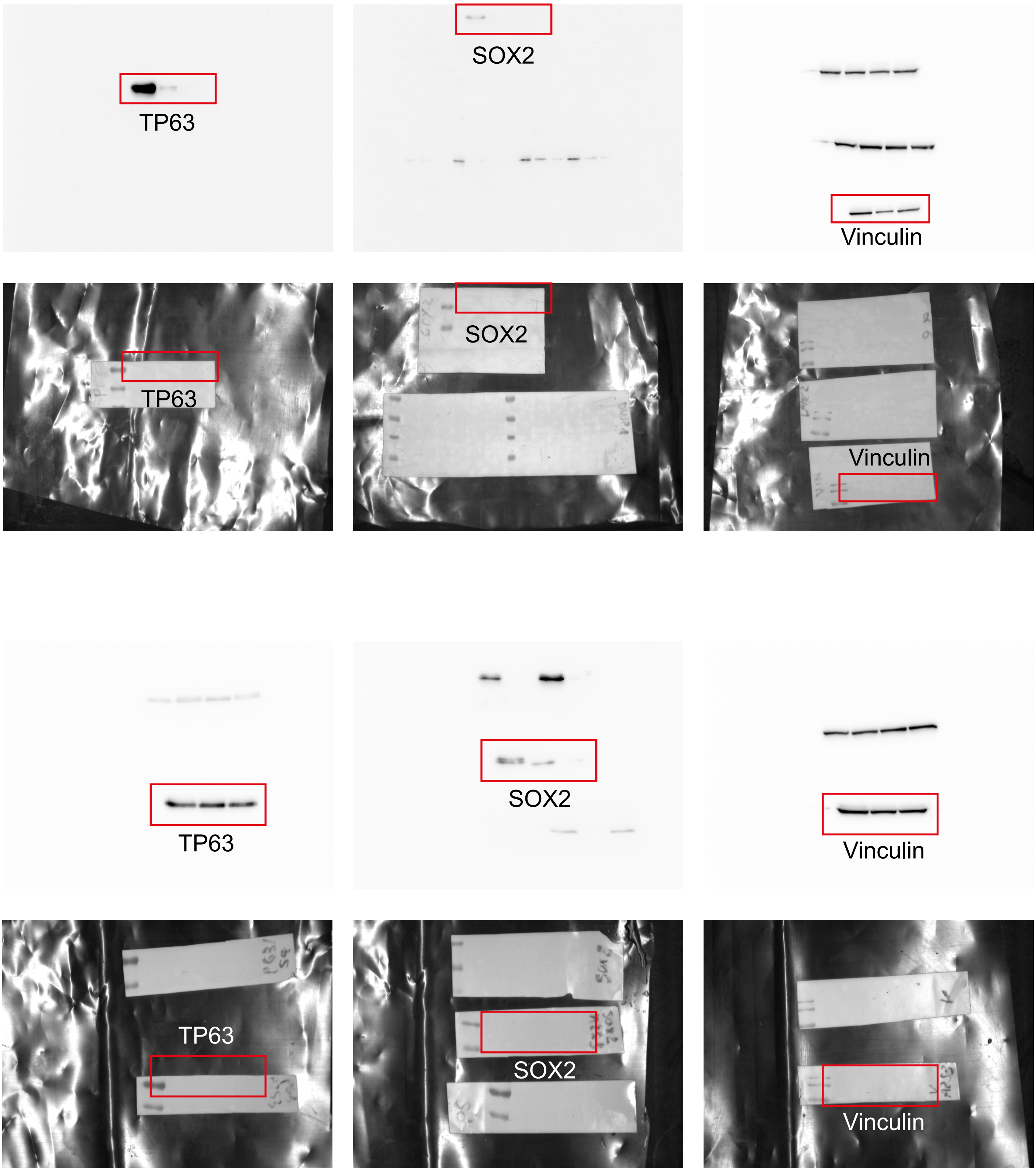

Supplement: Supplementary file 1 — Supplementary Figures. [file 41598_2024_65945_MOESM1_ESM.pdf]
